# A subtelomeric non-LTR retrotransposon *Hebe *in the bdelloid rotifer *Adineta vaga *is subject to inactivation by deletions but not 5' truncations

**DOI:** 10.1186/1759-8753-1-12

**Published:** 2010-04-01

**Authors:** Eugene A Gladyshev, Irina R Arkhipova

**Affiliations:** 1 Josephine Bay Paul Center for Comparative Molecular Biology and Evolution, Marine Biological Laboratory, 7 MBL Street, Woods Hole, MA 02543, USA; 2 Department of Molecular and Cellular Biology, Harvard University, Cambridge, MA 02138, USA

## Abstract

**Background:**

Rotifers of the class Bdelloidea are microscopic freshwater invertebrates best known for: their capacity for anhydrobiosis; the lack of males and meiosis; and for the ability to capture genes from other non-metazoan species. Although genetic exchange between these animals might take place by non-canonical means, the overall lack of meiosis and syngamy should greatly impair the ability of transposable elements (TEs) to spread in bdelloid populations. Previous studies demonstrated that bdelloid chromosome ends, in contrast to gene-rich regions, harbour various kinds of TEs, including specialized telomere-associated retroelements, as well as DNA TEs and retrovirus-like retrotransposons which are prone to horizontal transmission. Vertically-transmitted retrotransposons have not previously been reported in bdelloids and their identification and studies of the patterns of their distribution and evolution could help in the understanding of the high degree of TE compartmentalization within bdelloid genomes.

**Results:**

We identified and characterized a non-long terminal repeat (LTR) retrotransposon residing primarily in subtelomeric regions of the genome in the bdelloid rotifer *Adineta vaga*. Contrary to the currently prevailing views on the mode of proliferation of non-LTR retrotransposons, which results in frequent formation of 5'-truncated ('dead-on-arrival') copies due to the premature disengagement of the element-encoded reverse transcriptase from its template, this non-LTR element, *Hebe*, is represented only by non-5'-truncated copies. Most of these copies, however, were subject to internal deletions associated with microhomologies, a hallmark of non-homologous end-joining events.

**Conclusions:**

The non-LTR retrotransposon *Hebe *from the bdelloid rotifer *A. vaga *was found to undergo frequent microhomology-associated deletions, rather than 5'-terminal truncations characteristic of this class of retrotransposons, and to exhibit preference for telomeric localization. These findings represent the first example of a vertically transmitted putatively deleterious TE in bdelloids, and may indicate the involvement of microhomology-mediated non-homologous end-joining in desiccation-induced double-strand break repair at the genome periphery.

## Background

Mobile genetic elements are divided into two types according to their mode of transposition: retrotransposons, which require an RNA intermediate to synthesize a new copy with the aid of the element-encoded reverse transcriptase (RT), and DNA transposons, which do not require an RNA intermediate for transposition. Retrotransposons, in turn, are divided into two large classes according to the presence, or lack, of long terminal repeats (LTRs): LTR retrotransposons are framed by LTRs, while non-LTR retrotransposons are not (reviewed in [1-3]). When cDNA synthesis is primed extrachromosomally, template jumps during reverse transcription lead to the formation of LTRs. In contrast, if cDNA synthesis is primed directly at the insertion site by the 3'OH at the nick in chromosomal DNA (target-primed reverse transcription, or TPRT), no LTRs are formed. The nick is introduced by the non-LTR retrotransposon-encoded endonuclease (EN), which may or may not exhibit insertion preferences. RT then uses the endonuclease-generated 3' hydroxyl to prime cDNA synthesis and is believed to be highly prone to premature termination of reverse transcription, which results in formation of numerous 5' truncated copies of non-LTR retrotransposons (often called 'dead-on-arrival') [4-6]. Typically, while the overall copy number of non-LTR retrotransposons in eukaryotic genomes tends to be high rather than low, there usually exist relatively few master copies which have the capacity to give rise to the new copies [7,8]. These master copies, however, need to persist in their corresponding host genomes for extended evolutionary times, as horizontal transfer of non-LTR elements is believed to be exceptionally rare [1].

Bdelloid rotifers are small freshwater invertebrates with the ability to reproduce entirely asexually and to undergo cycles of desiccation and rehydration at any stage of their life cycle. These features may be related to their peculiar genome structure: bdelloids are degenerate tetraploids, with chromosomes present in quartets, each comprising two co-linear pairs, with only a minority of genes common to both pairs, in the same order and orientation [9,10]. Gene copies from different co-linear pairs exhibit very high levels of divergence, which was initially interpreted as inter-allelic divergence accumulated following an ancient loss of sex [11] but which, in fact, reflects the divergence between homeologs. Variable and much smaller levels of divergence within a co-linear pair (0%-6%) presumably reflect the occasional operation of homogenizing processes such as gene conversion and mitotic crossing-over. The extraordinary resistance of bdelloid rotifers to ionizing radiation may have evolved as an adaptation to frequent desiccation/rehydration cycles to protect the genomes from DNA damage [12].

Retrotransposons in bdelloid rotifers, including non-LTR retrotransposons, have remained elusive for some time. Initial screens employing degenerate polymerase chain reaction (PCR) primers targeted to multicopy LINE-like and *gypsy*-like elements turned up negative, despite yielding positive results in 39 diverse species from 23 animal phyla [13]. DNA transposons, however, were easily detectable even in early PCR screens, and, like in other species, exhibited patchy distribution, in agreement with their ability to transfer laterally and to evolve *via *multiple rounds of invasion, amplification, decay and horizontal escape [13,14]. The presence of vertically-transmitted non-LTR elements, however, might pose a problem in asexual species, which could eventually be overcome by the load of deleterious mutations, lacking the capacity to get rid of harmful transposable element (TE) insertions *via *meiotic recombination [15].

Analysis of about 1.5 Mb of gene-rich DNA from two bdelloid species, *Adineta vaga *and *Philodina roseola *[9,10] (JL Mark Welch, personal communication) also failed to reveal the presence of mobile elements, either intact or decayed. Several 40 kb - 70 kb co-linear contigs including *hsp82*, histone, *Hox *genes, and their genomic environment, were obtained by sequencing of overlapping fosmid library clones and contained only a single large indel polymorphism which was tentatively ascribed to a foldback-like DNA TE insertion [14]. The overall gene density, however, is quite high, with coding sequences occupying about 50% of genomic DNA (see Figure Three in [16]). Such a conspicuous lack of mobile DNA in gene-rich regions of the genome is quite intriguing, since genomic DNA from gene-rich regions - even of those model eukaryotes which are regarded as relatively TE-poor - contains, on average, 7.7-12.3 retrotransposons and 2.3-3.6 DNA TEs per Mb (*Drosophila melanogaster *[17]), or ~ 7 retrotransposons and ~ 19 DNA TEs per Mb (*Caenorhabditis elegans *[18]).

We were able, however, to find genomic regions which do not appear refractory to TE insertion, but, in contrast, are highly enriched in TEs. Our efforts aimed at cloning and sequencing telomeres, which are a lot less conserved than the core genome and are typically rich in repetitive and mobile DNA, revealed several types of TEs inhabiting bdelloid chromosome end regions. These included *Athena *retroelements specialized for terminal transposition [19], low copy-number retrovirus-like elements *Juno *and *Vesta *[20], numerous DNA TEs of various kinds [14] and R9 insertions into 28S ribosomal genes [21]. In the present study, we describe a non-LTR retrotransposon belonging to the jockey clade, which may be (or has recently been) active, is located preferentially in subtelomeric regions and is characterized by several unique features such as the lack of 5' terminal truncation and a high frequency of internal deletions associated with microhomologies.

## Results

### Structural organization and copy number

**Figure 1:**
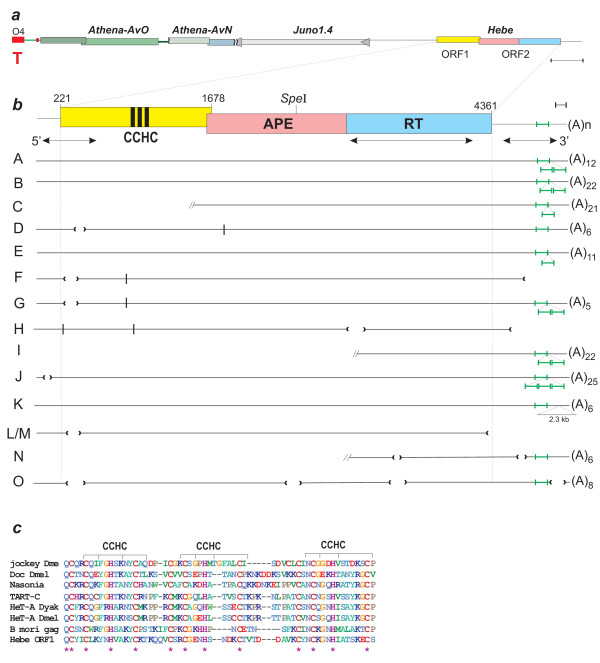
**Structure and polymorphism of the *Adineta vaga Hebe *retrotransposon**. (a) Genomic environment of the subtelomeric *Hebe *copy (K in panel b), including two head-to-tail *Athena *retroelements and a retrovirus-like long terminal repeat (LTR) retrotransposon *Juno*. The region containing *Athena *elements has likely been acquired from another telomere, O3 (reference [19]), possibly by break-induced replication. T, telomeric repeats. Scale bar, 1 kb. (b) Structure of a full-length *Hebe *consensus copy, and alignment of genomic copies to the consensus. APE and RT denote the AP-like endonuclease and RT domains, respectively; vertical black bars denote the three CCHC zinc knuckle motifs; (A)_n_, poly(A) stretch;//, copies that were truncated by cloning and could not be sequenced to completion. Deletions are indicated by brackets; in-frame stop codons, by vertical lines; probes used for library screening, by double-headed arrows; 72-bp tandem repeat units in the 3' untranslated region are in green. Also shown are the base coordinates for the start and stop of open reading frame (ORF) in the consensus sequence and the position of the unique *Spe*I site. Scale bar, 0.1 kb. (c) Three zinc knuckle motifs in *Hebe *ORF1 (panel a) and comparison with selected non-LTR elements from Figure 4b. Highly conserved residues are designated by asterisks.

In an extended genome walk directed from the chromosome end inwards, one of the telomeres from the bdelloid rotifer *A. vaga *(telomere O.4; [19]) was found to carry a very long chain of telomere-associated retrotransposons (EF485020; Figure 1a). In addition to two consecutive *Athena *retroelements, the most proximal of which was 3'-truncated by fusion with the oppositely-oriented retrovirus-like LTR retrotransposon *Juno*, the head-to-tail retrotransposon chain continued with a non-LTR retrotransposon encoding two open reading frames (ORFs). The first ORF contained three Zn-knuckle motifs (Figure 1a and 1c; Additional File 1) and appeared most similar to the *gag*-like ORFs from two *Drosophila *telomere-associated retrotransposons, *TART *and *HeT-A*, while the second ORF had homology to the apurinic/apyrimidinic (AP) endonuclease and RT domains from other representatives of the jockey clade, with the highest degree of similarity to ORF2 of the retrotransposon *Syrinx *from the putatively asexual ostracod crustacean, *Darwinula stevensoni *[22]. We named this element *Hebe*, as it appeared to be relatively young and capable of giving rise to new copies.

In order to determine the exact boundaries of the *Hebe *element, we designed a 1-kb PCR probe spanning the entire core RT domain (Figure 1b) and used it to screen the *A. vaga *genomic library to obtain additional *Hebe *copies. A second 0.6-kb probe spanning the 3' untranslated region (UTR) was also used in subsequent library screens in order to find out whether *Hebe *might give rise to a large number of short 3' truncated copies which would have been missed by the RT probe. Interestingly, hybridization with the second probe yielded few, if any, additional hybridizing spots, indicating a lack of copies which are 5' truncated in the region between the two probes. The number of fosmids obtained from screening *~ *4 *A. vaga *genome equivalents is shown in Table 1.

**Table 1:** Characteristics of *Hebe *copies.

Copy	No. of fosmids	Stop codon	5' truncation	3' truncation	Internal deletion	Genomic environment
**A**	4	-	-	-	-	Tandem repeats
**B**	2	-	-	-	-	Leucine rich repeat protein
**C**	1	?	?	-	-	No significant hits
**D**	2	+	-	-	-	DNA TE, piggyBac-like
**E**	3	-	-	-	-	No significant hits
**F**	6	+	-	+	+	Tandem repeats (=G)
**G**	6	+	-	-	+	Tandem repeats (=F)
**H**	2	+	-	+	+	TPR protein
**I**	4	?	?	-	-	Kelch repeat; Fungal ORF
**J**	3	-	-	-	+	TPR repeat protein
**K**	2	-	-	+	-	Telomere; telomeric repeats
**L**	2	-	-	+	+	LTR TE (=M)
**M**	1	-	-	+	+	LTR TE (=L)
**N**	1	?	?	-	+	No significant hits
**O**	4	-	-	+	+	Telomeric repeats

See Additional File 2 for sequences of copies A-O.

**?**, copies truncated by cloning.

TE, transposable element; TPR, tetratricopeptide repeat; ORF, open reading frame; LTR, long terminal repeat.

All hybridizing fosmids were first sequenced with the primer located at the C-terminus of ORF2 and directed outwards, in order to find out how many independent insertions (flanked by differing genomic sequences) can be identified on these fosmids. The sequences fall into 13 groups defined by the adjacent flanking regions, which correspond to 13 independent insertion events. One group (L/M) consists of two subgroups which share the same deletions and flanking sequences, but differ by six point mutations, indicating that one was recently copied from the other (for example, in the course of segmental duplication, break-induced replication or gene conversion between two members of a co-linear pair). Another group (F/G) shares the 5' flank and 100% identity in sequence, but differs by a deletion involving the 3' end of copy F. Eleven of the 15 sequenced copies do not exhibit 3' truncation and have a characteristic poly(A) tail, which varies in length between 5 and 25 nucleotides and is located 11 bp downstream of the AATAAA signal. A peculiar feature of the 3' UTR is the presence of a 72-bp tandem repeat, the copy number of which varies from 1 to 4 between different copies, yielding variation in the 3' UTR length between 0.8 and 1 kb (Figure 1b).

**Figure 2:**
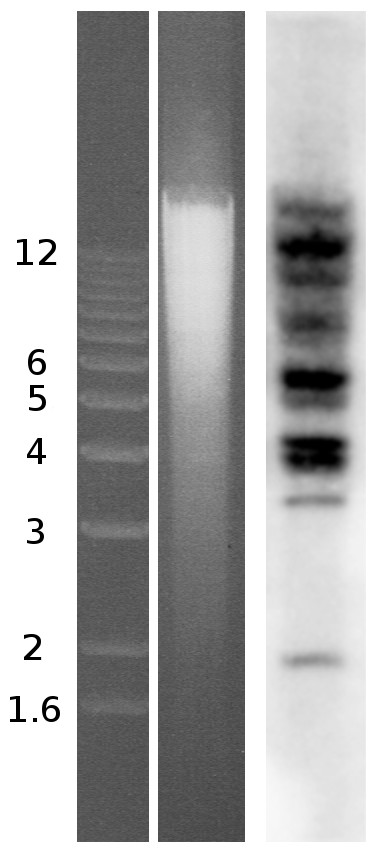
**Southern blot analysis of *Hebe *genomic copies**. Genomic DNA from *Adineta vaga *was digested with *Spe*I/*Sac*II (centre lane) and hybridized with the RT probe (right lane). Marker sizes in kbp are indicated on the left.

In order to obtain an independent estimate of the copy number, we performed a Southern analysis of *A. vaga *genomic DNA digested with restriction endonucleases *Spe*I and *Sac*II. The latter does not have a recognition site in any of the sequenced *Hebe *copies, while the former cuts only once (Figure 1b). We used two enzymes to achieve a better resolution of the individual bands on the gel, by digesting away larger amounts of flanking sequences. The use of the 1-kb probe spanning the RT domain yielded a set of bands corresponding to each genomic *Hebe *copy plus variable amounts of adjacent flanking sequences (Figure 2). The results are in excellent agreement with the estimates obtained from genomic library screening: there are 11 hybridizing bands on the gel, four of which are of double intensity, therefore yielding a total of 15 different copies.

### Preference for subtelomeric regions and lack of 5' truncations

The number of fosmids in each group, corresponding to a single genomic copy, turned out to be lower than that expected on the basis of genome coverage (typically between 1 and 4, with the exception of groups F/G) (Table 1). In comparison, for the same membranes, the corresponding number of fosmids carrying the single-copy *hsp82 *gene was between four and six for each haplotype [10,23] and the number of fosmids carrying the histone cluster was similarly high (five to seven for each haplotype) [24]. Such under-representation is highly indicative of subterminal localization of the *Hebe*-containing fosmids, which would be present in the genomic library in lower numbers as the size-selection step in the library construction protocol puts the terminal regions at a disadvantage (see [19]).

Another line of indirect evidence pointing at localization in subterminal regions is the nature of the surrounding flanking sequences: fosmid end-sequences and the immediately adjacent genomic flanking regions are characterized by features previously found in other bdelloid telomeric fosmids, such as tandem repeats, other TEs, ORFs coding for proteins of repetitive nature or of foreign origin and short stretches of telomeric repeats (Table 1). Finally, telomeric retrotransposons often possess 3' UTRs which are prone to formation of tandem repeats [25,26] and this is also the case for the *Hebe *element (Figure 1b; Additional File 2).

We also sought to confirm that the 5' and 3' ends of *Hebe *are equally represented in the genomic library, as was indicated by our initial screening. To this end, we probed two additional membranes with the 5' and 3' *Hebe *probes of approximately equal length (Figure 1b) and counted the number of hybridizing spots: a total of 101 spots were shared between the two probes; the 5' probe revealed 15 additional spots not detected by the 3' probe; and the 3' probe revealed 26 additional spots not detected by the 5' probe. Thus, there is no significant excess of *Hebe *copies containing only the 3' end and additional spots in both directions can be explained either by deletions involving one of the termini, as seen in Figure 1b, or by the presence of incomplete copies truncated by cloning.

### Divergence between copies

**Figure 3:**
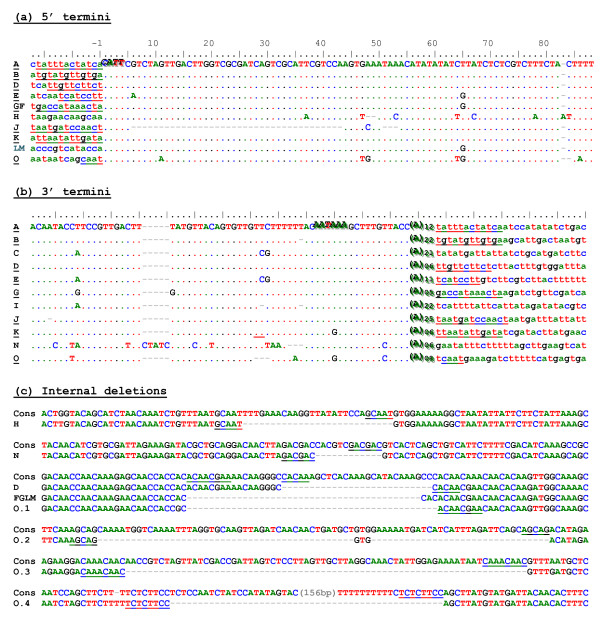
**Sequences of *Hebe *5' and 3' termini and internally deleted regions**. (a) 5'-terminal regions from 12 sequenced copies; (b) 3'-terminal regions from 11 sequenced copies, designated as in Figure 1b. Copies represented in both a and b sets, and the corresponding target site duplications, are underlined. The CATT initiator sequence and the polyadenylation signal are italicized. (c) Comparison between the *Hebe *consensus sequence (Cons) and the corresponding deleted regions from copies with internal deletions shown in Figure 1b. Copies G, F, L and M share the same deletion boundaries. Four deletions in copy O (O.1, O.2, O.3, O.4, from left to right) are shown. Microhomologies are underlined.

In order to evaluate the intactness of *Hebe *copies and the degree of divergence between them, we sequenced these copies by primer walking. The results are shown in Figure 1b (see also Additional File 2). *Hebe *exhibits a number of peculiar features which are not in agreement with the currently prevailing views on proliferation of non-LTR retrotransposons. First, we could not find any copies exhibiting 5'-terminal truncation, which normally results in formation of a large number of inactive copies and is believed to occur due to the premature dissociation of RT from its template. Second, it is evident that inactivation of individual copies occurred mostly *via *deletions. Nine copies (D, F/G, J, H, L/M, N, O) carried internal deletions 12-200 bp in length affecting the integrity of their ORFs, and comparison of the sequences at deletion boundaries (Figure 3c) reveals that most of them contain characteristic microhomologies (5-14 bp), which are typically regarded as a hallmark of non-homologous end-joining events resulting in imprecise repair of double strand DNA breaks (DSBs) (reviewed in [27]). Four copies (F, H, L/M) exhibit 3' terminal truncation, which could have also occurred by deletion, although in this case it is not possible to compare the sequence with its original non-deleted version to reveal the presence of microhomologies. Copies D, F and G carry in-frame stop codons. Copy K contains a 2.3-kb insertion of unknown nature 26 bp upstream from the poly(A) tract. Overall, three copies (A, B and E) may be considered intact, because they carry no obvious defects in their ORFs and possess intact 5' and 3' termini. These copies are flanked by 7-12 bp target site duplications (Figure 3a and 3b) and differ from each other by 29-33 nucleotide substitutions.

Notably, the *Hebe *element begins with the sequence CATT, which is the canonical initiator motif in many eukaryotes and is characteristic of internal RNA pol II promoters found in *Drosophila *non-LTR retrotransposons which ensure that a full-length copy does not lose its promoter after retrotransposition [28-30]. The 5' UTR is rather short, being only 220 bp in length. There are two more copies (C and I) which we could not sequence in its entirety because fosmids containing these copies were truncated by cloning, but they appeared to be intact in their sequenced part. If these copies are given the benefit of the doubt, this may bring up to five the number of potentially intact *Hebe *copies in the genome.

**Figure 4:**
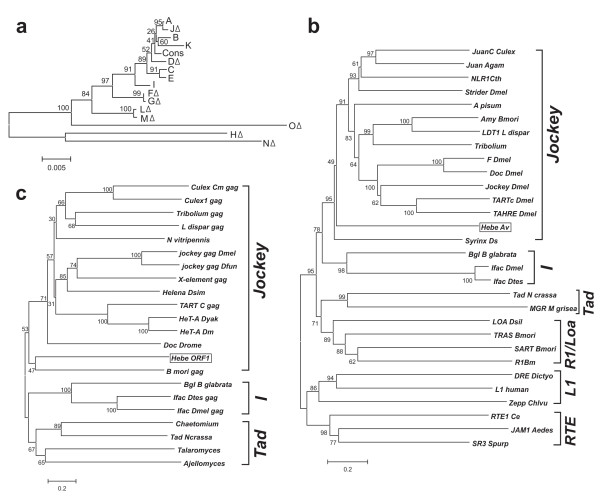
**Genealogy of *Hebe *genomic copies and phylogenetic placement of *Hebe *open reading frames (ORFs)**. (a) Neighbour-joining phylogram of 15 *Hebe *genomic copies (Table 1), designated as in Figure 1b, plus the majority-rule consensus sequence (Cons). Copies with deletions are indicated by Δ. Scale bar, nucleotide substitutions per site. (b, c) Neighbour-joining phylograms of (b) ORF2 from representatives of major non-long terminal repeat (non-LTR) retrotransposon clades, combining EN and RT domain sequences about 900 amino acids in length and (c) ORF1 coding for *gag*-like proteins from diverse non-LTR retrotransposons, about 500 aa in length. Clades are designated with square brackets. Scale bar, amino acid substitutions per site. Bootstrap support values from 1000 replications are indicated at the nodes.

The genealogy of *Hebe *genomic copies is depicted in Figure 4a. Overall, this pattern is characteristic for non-LTR elements, with inactive copies yielding long terminal branches, as they accumulate numerous point mutations in addition to deletions and the potentially active elements yielding much shorter branches, which are indicative of relatively recent activity. Pairwise all-by-all comparison of ORF1 and ORF2 coding sequences reveals an overall excess of synonymous substitutions over non-synonymous ones, which gradually fades away as copies become more decayed (Additional File 3). Again, this is in agreement with relatively recent activity of the element, although no two independent insertions were found which differed by less than 10 nucleotide substitutions (copies A+J and C+E, which potentially represent the most recent retrotransposition events; F/G and L/M are not independent insertions).

### Similarities to other retrotransposons

Phylogenetic analysis of the 896-aa ORF2 (including EN and RT domains) places *Hebe *as a basal member of the jockey clade of non-LTR retrotransposons, which also includes *TART *and *HeT-A/TAHRE *(Figure 4b), while its closest known RT relative is the *Syrinx *element from *D. stevensoni *[22]. The 485-aa ORF1, which codes for a *gag*-like protein with three zinc knuckle motifs (CCHC; Figure 1b and 1c), is expected to evolve faster than ORF2 and, indeed, it exhibits much lower levels of sequence identity (≤25%, compared to 34% for RT and 32% for EN) to *gag*-like proteins from other non-LTR retrotransposons (Figure 4c). It is intriguing that the top BLASTP hits included the corresponding *gag*-like ORFs of *Drosophila *telomere-associated retrotransposons, some of which have evolved special properties targeting them to telomeric heterochromatin [31]. However, telomeric targeting is known to have evolved independently in members of other non-LTR clades such as R1 [32].

## Discussion

In this study, we report, for the first time, a non-LTR retrotransposon in the genome of a bdelloid rotifer *A. vaga*, which exhibits characteristics of an active (or recently active) vertically-transmitted retrotransposon without apparent preference for a specific target sequence and may contribute to understanding the reasons behind the conspicuous lack of TE insertions in the gene-rich regions of bdelloid genomes [16]. It appears that, despite relatively recent activity, the element has not reached high copy numbers and most of its copies are riddled by deletions. In addition, the majority of insertions appear to be concentrated in subtelomeric regions. We believe that we were able to clone and sequence most, if not all, of the genomic copies. Even though two sequence variants (C and N) were found in the library only once, and it is formally possible that a few telomere-proximal copies were not represented in the library, the Southern blot analysis is in good agreement with our original estimates from library screening and indicates that it was exhaustive.

Concentration near telomeres may have two possible explanations, which are not necessarily mutually exclusive: either the element preferentially inserts into subtelomeric regions (for example, after having developed an affinity to certain epigenetic marks in subterminal chromatin) or it inserts randomly throughout the genome, but insertions in gene-rich regions are eliminated by selection against deleterious effects of such insertions on nearby genes and/or against deleterious chromosomal rearrangements caused by ectopic recombination between insertions. A currently, or recently, active non-LTR retrotransposon can be expected to serve as a good model system with which to discriminate between these two possibilities, because any short 5'-terminally truncated insertions would have had a better chance of being found near genes, as they would constitute less efficient targets for ectopic recombination events [33,34]. However, these predictions could not be fulfilled because, surprisingly, we were unable to find any 5' truncated retrotransposed insertions in an exhaustive screen of the genomic library. If the element's RT is not at all prone to premature termination of cDNA synthesis, its chances of survival may increase if there is any insertional specificity disfavouring insertion into gene-rich regions by recognizing certain chromatin features. Although the molecular determinants for telomeric targeting by *gag*-like proteins in *Drosophila *are not known, the similarity between ORF1s from organisms as distant as fruit flies and rotifers is intriguing. While telomeric targeting may be a possibility, the paucity of TE insertions in gene-rich regions most probably results from synergistic selection against TE-mediated deleterious rearrangements following DSB repair (see [12,35]).

While we have previously observed little or no deletions in other telomere-associated TEs, most *Hebe *copies contain inactivating deletions, apparently formed *via *joining of microhomologies in the vicinity of a DSB. Bdelloids are known for their ability to survive multiple rounds of desiccation and rehydration [36] and for their extraordinary resistance to ionizing radiation [12], which is accompanied by extensive DNA breakage and rejoining and has likely evolved as an adaptation to the desiccation-prone bdelloid lifestyle. DSB repair in bdelloids most likely occurs by homologous repair, which does not leave lesions in DNA. Indeed, examination of >1 Mb of gene-rich co-linear pairs of bdelloid genomic DNA does not reveal any molecular footprints of non-homologous end joining (NHEJ) repair events. Repair, however, could also occur by error-prone NHEJ (also called microhomology-mediated end joining or MMEJ), which would seal the break after resection using short microhomologous stretches of DNA in the vicinity, resulting in deletion of the intervening DNA sequence (reviewed in [27]). Deletions in *Hebe *copies were likely formed by this mechanism. It should be noted that similar deletions were previously seen in non-LTR retrotransposons of *Giardia*: of three retrotransposon families, one was preferentially disrupted by microhomology-mediated deletions [26]; members of this family, *GilD*, are found in gene-poor genomic regions next to variant-specific surface proteins. We also observed microhomology-mediated deletions in two out of a few dozen PCR-amplified fragments of *A. vaga mariner *DNA transposons, although in this case their chromosomal location was unknown ([14] and I R Arkhipova, unpublished data). Such deletions are not uncommon among TEs ([5]; reviewed in [37]) and tend to be correlated with heterochromatic environment (see [15] for discussion).

We hypothesized that, in otherwise asexual bdelloid populations, genetic exchange might take place without conventional meiotic sex, based on the observation that the bdelloid germ line is susceptible to invasion of foreign DNA, accumulated mostly at telomeres [16]. Such penetrability of the germ line could potentially allow any lost TEs to be regained from bdelloid DNA released into the environment, even in the absence of meiosis and syngamy. Alternatively, the presence of non-LTR elements may constitute evidence of a cryptic sexual process occurring in bdelloids. Further investigations into the mechanisms by which bdelloids combat repetitive elements and repair their DNA, as well as comparative analyses of bdelloid whole-genome sequences and identification of additional non-LTR retrotransposon families, may be expected to shed more light on the remarkable compartmentalization of bdelloid TEs.

## Conclusions

The non-LTR retrotransposon from the bdelloid rotifer *A. vaga*, named *Hebe*, was found to undergo frequent microhomology-associated deletions, rather than 5'-terminal truncations characteristic of this class of retrotransposons. In combination with the tendency for telomeric localization, these findings may indicate the involvement of the MMEJ pathway in the repair of double-strand breaks at the genome periphery and may eventually help to explain the overall under-representation of TEs in the bdelloid core genomic regions and their abundance at telomeres. It remains to be seen whether the presence of vertically-transmitted TEs in bdelloids may be indicative of sexual exchange.

## Methods

### Library screening and fosmid analysis

The *A. vaga *genomic fosmid library [23] was screened with the ^32^P-labelled 1-kb RT domain fragment amplified by PCR using a pair of primers F1 (CCAGTGGTTTGATGATGGTGT) and R1 (CTGCTGATACGTTGCCACTTC), and the 0.6-kb 3'UTR fragment amplified with primers 3'UTR-F1 (ATGTCACATACAATCCAGCTTC) and 3'UTR-R1 (GTAACATAAAGTCAACGGAAGG). Selected fosmids were end-sequenced with standard T7 and ccFos primers, split into different groups with the primer seq1 (CAACAAACAACGACATTACACTG) directed into the flanking host sequences, and several fosmids from each group were sequenced by genome walking with custom primers (F1; R1; seq3, AGCCTTTTTCCACATTGCTGG; seq 4, AAAGTTGGACTATCATCTTCG; seq5, GTTGGTGCAAGTCATGGAAAT; seq6, TCGATCTTCTTGATCTTCTGATG; seq7, TGTCATGGATATTGACTTCAGCA). The 5' probe for additional membrane screening to compare representation of 5' and 3' ends was obtained using primers 5p (GATCAGTCGCATTCGTCCAA) and seq7, and the 3' probe - using primers seq1 and 3'UTR-R1. Entire fosmid sequences were obtained by shotgun subcloning into pBluescript II SK- and sequenced on the ABI3730XL at the W M Keck Ecological and Evolutionary Genetics Facility at the Josephine Bay Paul Center for Comparative Molecular Biology and Evolution, Marine Biological Laboratory. Sequences were deposited in GenBank under accession numbers EF485020 and GU176366-GU176379.

### Southern blotting

*A. vaga *genomic DNA was sequentially digested with restriction endonucleases *Spe*I and *Sac*II, fractionated on 0.7% agarose gel, and transferred to Hybond+ membrane (Amersham). The RT probe (about 1 kbp) was amplified from *A. vaga *genomic DNA using the primer pair F1/R1 described above, gel-purified using the Qiagen Gel Extraction Kit and labelled with ^32^P-dCTP using random primers (Invitrogen, CA, USA). The probe was hybridized at high stringency (2×SSC, 65°C overnight).

### Phylogenetic analysis

Alignment (ClustalW) and phylogenetic analysis was done with MEGA4 [38], using either nucleotide sequences (maximum composite likelihood; pairwise deletion; 1000 bootstrap replications) or amino acid sequences (neighbour-joining or minimum evolution; Poisson correction or *P*-distance; pairwise deletion; 1000 bootstrap replications). Amino acid sequence alignments in BoxShade format are presented in Additional File 1. Pairwise Ka/Ks ratios were calculated by the program DIVERGE from the Wisconsin package (Accelrys Inc., San Diego, CA, USA).

## Abbreviations

DSB: double-strand DNA break; LTR: long terminal repeats; MMEJ: microhomology-mediated end joining; NHEJ: non-homologous end joining; ORF: open reading frame; PCR: polymerase chain reaction; RT: reverse transcriptase; EN: endonuclease; TE: transposable elements; TPRT: target-primedreverse transcription; UTR: untranslated region.

## Competing interests

The authors declare that they have no competing interests.

## Authors' contributions

EG and IA designed and performed experiments and analysed the data. IA wrote the manuscript. Both authors read and approved the final manuscript.

## Supplementary Material

Additional file 1 BoxShade alignment of amino acid sequences from the most conserved regions of open reading frame (ORF) 1 (p.1), endonuclease (p.2), and reverse transcriptase (p.3) domains from selected non-long terminal repeat retrotransposons analysed in Figure 4. Click here for file

Additional file 2 Nucleotide sequences of *Hebe *elements obtained in this study. Click here for file

Additional file 3 Analysis of non-synonymous to synonymous substitution ratios in open reading frame (ORF) 1 and ORF2 of *Hebe*. Click here for file
