# Supplementary material for: A subtelomeric non-LTR retrotransposon Hebe in the bdelloid rotifer Adineta vaga is subject to inactivation by deletions but not 5' truncations
Source: Mob DNA. 2010 Apr 1;1:12. doi: 10.1186/1759-8753-1-12 (PMC2861651; doi:10.1186/1759-8753-1-12)
Supplement: Additional file 1 — BoxShade alignment of amino acid sequences from the most conserved regions of open reading frame (ORF) 1 (p.1), endonuclease (p.2), and reverse transcriptase (p.3) domains from selected non-long terminal repeat retrotransposons analysed in Figure 4. [file 1759-8753-1-12-S1.PDF]

```
jockey_gag_Dmel    1 LLSAEAEN-MEQDVSDADSD-IEDSAARDGGGQSAK-YSKPPAICVPSVSD-----PVTLERALNLSTG--SSNYYIRIS--RFGVSRIYTANPDAF---RTAVKELNKLNCQFWHHQLK--------EE
jockey_gag_Dfun    1 ALALEADNVMEQDLAENCSNGEEDVPVVNMNRQSTNGPSKPPAICVPNVDN-----PHSLEQALNSCVG--DNSYSIRTS--KFGVSRIYTINADAF---RAVVKHLTSLNCQFWHHQLR--------ED
X-element_gag      1 -EIGDLYDQPSTSHQAAIAAAKRDAASAGTTSSAKRAQSKPPPIVMEGVDD-----VYLMMQSIENIVD--LEKIEARAS--MSGVLRLYAADANTF---RTIVNWLEIEEYEFHCYQLK--------ED
L_dispar_gag       1 ATTATAAPAPPVVSQAALTAAPPPTDSNTVAPPTVKKSLAPPPLYIRDKD-------KWLAISAWLNTN--RISYKSAKS--TPQGIRVDLHSSADH---RSVSKMLGNQQIPYHTYTLP--------EA
Tribolium_gag      1 SPPGLRPDSPPGLHPGSPPGLRPDSPPPQDSAPTAPKASRIPPIFLRDAG------KWHLVSQKAN--------FTKARS--VSDQIRIQPTTVADF---RLFTRFMEAERIPFHTFTLP--------EE
Culex1_gag         1 EQGGDQNSLPSIPTAP---------PVLKIGEPTKAKKVRIPPISVVGKSTRQ---LREFLGKSNIA----QTAYNMKAT---KSGVQLLCSGDDSF---RGAVRALRTANIEFHTYTPA--------AE
Culex_Cm_gag       1 DEENNNNTDGSTTTDDDDDDGRARKKVPTPKKNNSPTERRPPPIFVLDTLADD---VDKLLEG---------LGYCLKIG---ESAVQVITLNKKSF---DLVFEELKRSNFNFYTFNPE---------K
Helena_Dsim        1 ASTSCASAAPNIPNDDCEPNVTSNSPQQYTDKQNSYKQSKPPQIVLS--LTN----LNDLYELITEVTS--LDNLTVKVN--QGETVRILPKDSDTC---RAIVNLFVNSGIEFHTYQMK--------EE
Doc_Drome          1 NNGNENSAVNNSNRYAILADSATEQPNEKTVGE--PKKTRPPPIFIREQSTN--ALVNKLVALIGDSKF---HIIPLKKG--NIHEIKLQIQTEADH---RIVTKYLNDAGKNYYTYQLK--------SC
N_vitripennis      1 SQRKRRRTGARIVPKSAKFLSESESNSESSDSEIETAKAKPPPILIREEDR-----WPKI-QRALAEHG---IQLRRAVN--LSDGVKIYPETVDSF---REISKKLDSSEIQYTTYQLK--------QD
TART_C_gag         1 --SLQKSAEMDTAP--AISPSNTAADSDLPPWKTVPASRKPPSIFLSNIQQ-----IIPLIEKLNYKAG--VNSFTTKSE--LGNNIRIQAKTMDAH---NAIQNVLLEANIPLHSHQPK--------SA
HeT-A_Dyak         1 --SEKASARSVPAQTEIAPPPPKNSATELPPWQIVPQSRRAPPIHIKNVRE-----IVPLLEKLNYSAG--VDSFTTKTS--IGNGVTIQAKDLTAH---RIIKDILAKSGIPYYSNQNK--------SE
HeT-A_Dm           1 -CSINASARSAAAPPGIAPLPPHNTDAELPPWKIVPQSRRAPPILVNDVKE-----IVPLLEKLNYTAG--VSSYTTRAI--EGNGVRIQAKDMTAY---NKIKEVLVANGLPLFTNQPK--------SE
Hebe_ORF1          1 --SIQ-SPQRTTQDGKAGPYALKSSQPTFSIAPLILEGVNLNKLQLNDILKQ----HLAEVNIHDIQLG--RNGIFTLYA--RDVKSFNNILNDFSS---ILSSNGQPSATVYVPRSIQRI-------KD
B_mori_gag         1 NKSGEVEEEDLMETEYNIRQNEENEEDWTVVKRSKAKRWKPNSVDVCISSKNQLPKQFALAKLFHRLNI--GGITRVKYI--NAFKVLLAFEDIAF----SEKFAACPEIQEQGWRIQNSW-------EV
Ifac_Dmel_gag      1 ----------MTDPPNIYKITSKTYQSQLGEPKFIIIKRNDNNSFERTS-------PFIIKKSVDFACG--GEVEGCKRT--RDGNLLIKTKNEL---------QARKLLKLTKIADED-----------
Ifac_Dtes_gag      1 ----------MSRPPDP-KLLTQWDKTTFLEPKYISINRKDNKSFENTS-------PFIIKKVIDYQCC--GEVDSVKKI--RDGSLLVKTKGTA---------QATRLMKITKFHDFE-----------
BgI_B_glabrata     1 KLLAPDLCLGKKRRIDDKKEEVPKSCATWP-SFLVVRCTEEGKSLTKLS-------PFIIYKGLKSVV---GEPKSVSKL-HKTMELLVEVDSKT---------HSDGLLRCRRLCDLQ-----------
Chaetomium         1 ATQGRSWAAVAAGPGQGLQAQPTKIIPQRADRELLVRGANVSADLAQRSPVE----IIQAINQASTKQGA----IAARK--LPSGDTVVTFNDSNTK------SWHSNNTQWIQQAFGEQAKE-----AG
Talaromyces        1 WKTVQYKKQALAPSKALSTTNLKPVSTRSKEERRLIFRRRYPKDAPTALKAD----ILLALNRALAKAGLPDFVRAVDSGYAASGALTVLLERGTRS-----STLVPVHNDTLLAAVRQTD--------P
Ajellomyces        1 APRTPPPAQTNSKEREIIVKLHNPEAAALLRDKTPGELRERVNNALKER--------TRSTERPIQV------VAAKQLK---SGDVSIHTVNIEDANKLREEQQWTQALGREAKALQPTYGVLVHSVCT
Tad_Ncrassa        1 DVVAKASPSKIGATLQQPPTTTKKFVPSNLERQLTIKGATIAAEFVNRSNEDTKTTLATCLGKKKPGLVVR-AATRMPT----TGDYVIVFDEPTR-------TWCWRNQAWAKEVFGPDAFI-----TM
consensus          1   ..                    .         . .  ..... .          .  ..  .    .     . ..      . ...           . .   .    . . ... .         .

jockey_gag_Dmel  108 KPYRVVLKGIHANVPSSQIEQAFS---DHGYEVLNIYCPRKSDWKNIQVNEDDNEATKNFKTR------QNLFYINLKQGP--NVKESLKITRLGRYR-VTVERA-TRRKELLQCQRCQIFGHS-KNYCA
jockey_gag_Dfun  111 KPYTVAVKGMHANVPKAQIEHAFA---DHGFEALNIYCPRKADWRNAQVSEDDDEATVNFKTR------QNMFFVNLKQGP--KIAEALKITQLGRYR-VTVERA-PRRREVLQCLRCHIFGHS-KNYCV
X-element_gag    110 RPYRVCVKGLHHSTLHHQIKDELE---KIGHKVLDIHTPLR---RNEPGTS--KASPVN------------MFFLNIAAAA--NNKEILAVKALCHMR-VVIEPL-RKRNAIVQCHRCQQIGHT-AKYCR
L_dispar_gag     109 KLLRAVIRNVPREIETKEILESL--------KTQDLPVVEVHRMIRGRGRYPLNMI-----------------LVCLTNNAE--GKGIFKIKTICGLSGVSVEPP-HKNGNLAQCHKCQLYGQS-SKNCF
Tribolium_gag    104 KTTRVVLRGIPVQVSTDEVFADL--------KRQGFNPISTHRMHT-----GKRQL-----------------PLVLLEAPLDQAKEVWKMKTVCSLM-VKVEKP-RKSGKAAQCHRCQRFFHA-QRNCT
Culex1_gag       101 QPMKVVLSGLPVYD-VDELETEL--------AGLGIHVSELKLFSRKVVGMEESAL----------------YLLHFPKGS-MKLADLQKIKAIFNIV-VRWRYFERKPIDAVQCHRCQRFGHG-MRNCN
Culex_Cm_gag     104 PPVKVVLQGYQDRP-ISDLKGHL--------SDAGIKPREIKVLSRKTTVTGTHTL----------------YLLYFDRGT-VKIQDLRRTKTLDGFW-VNWRFYTKNPTDVAQCHRCQKFGHG-SRNCN
Helena_Dsim      110 KPHRIVVKGPHHSTLTYEIIDNF--------KTYGFDVLQVHNPRSRRNREEKLNM----------------FFINIKTYA--KINDIYDINTICRQK-VRIERM-RKSSEIAQCIRCQEFGHT-AKYCR
Doc_Drome        111 KGLQVVLKGIEATVTPAEIIEAL--------KAKNFSAKTAINILNKDKVPQPLFKIE--------------LEPELQALKKNEVHPIYNLQYLLHRR-ITVEEP-HKRINPVQCTNCQEYGHT-KAYCT
N_vitripennis    109 RELIVVIRGVNESYSEEEILEEI-----------RHKHPAVKKVFRMRNGEKVWPL-----------------VIAHLDPSFAHAKTIFDLQTLGGLK-VTVEPK-RKSKFTPQCKRCQKFGHT-ANYCH
TART_C_gag       107 KGFQIVIRHLHQSTPTKWIESQL--------QDIGIATKFIRAMQFRDTRNPMRIH-----------------EVEVVPKADGSHLKVLLLKSLGGQT-VKVERK-RVSKDPTQCHRCQCFGHT-KNYCR
HeT-A_Dyak       109 RGFRVVIRHLHPSTPCSWITSEL--------QKLGHQTKFTRNMTNPATGGPMRMH-----------------EIEIVSAMDGSHLRILSIKQLGGQK-VEIERK-NRTRELVQCFRCQGFRHA-RNTCM
HeT-A_Dm         110 RGFRVIIRHLHHSTPCSWIVEEL--------LKLGFQARFVRNMTNPATGGPMRMF-----------------EVEIVMAKDGSHDKILSLKQIGGQR-VDIERK-NRTREPVQCYRCQGFRHA-KNSCM
Hebe_ORF1        110 TEKIAFVKRVDLELPNDRITEAL--------KNVGLEVTEVIRLTSKDGKTPTRTV-----------------KISFSDATN-RNIFVQTGLQVDCMH-FTAEPA-TQNSKPVQCYICLKYNHV-AKYCK
B_mori_gag       116 NISYGIVKNMEIGISEDDLMENI--------RTSNNCEIVAIKRLNKRNPASPGWI-----------------DSETIRLSF-KGNSLPEYVYLFNTR-VKVE-A--YIFPVTQCSNCWRFGHS-AKYCP
Ifac_Dmel_gag     90 ------VTASEHKTLN-FSKGVI--------YCNDLRHIDEDTILQELKPQKVSEVKKIMKRQNPNSNSDTNNITLVETGLIIITFESHKLPEIVRIG-YETVRVRDYIPLPLRCKKCLRFGHP-TPICK
Ifac_Dtes_gag     89 ------VTATEHKTLN-FSKGVI--------YSNDLRNIDIDEIKKELKPQKVIDATKIFKQKD---------NVLHETGLIIITFDTPTLPQHLMIG-YERVNVRPQTPRPLKCHNCQKYGHP-SKYCR
BgI_B_glabrata    99 ------VEVMPHKSLN-TSRGVI--------SSRDL--LECSEKEIVEGIEGVTHARRITRRRE---------GEEIKTATIILTFGTRTPPEYVKAG-YLRVPVRPYIPNPMRCFKCQGYGHG-AAVCK
Chaetomium       110 RTYAVLVKGLRKADLQGTTEEV-----------FGGELGLWLVDK-VKFRVPTNPGFTRA-------------TVLVALRDQEEARKACDQGIIWNAQLLDCQPY-WAMLEPKQCFKCWKWGHI-QRFCQ
Talaromyces      114 AVISVEISEQWHRVKVQAVPVDRYMYNDQ-----GLALAQEEIELGTPYRLKREPTWLKRAKTIQASNQ-RFATIVMTVGSLEEARTLINKGIKFGGRHHRVAPY-WESNPESICPRCCGIGHSGFMACG
Ajellomyces      114 DKENIDPSNQSRSIEKIQT--------ENATLHPGATITYVGWLTRTGAKKPTS-------------------SLVLEFTTKEHADRAIREGLVLDACYHHCELY-DRSCKLKQCYKCQKYGHI-GTQCN
Tad_Ncrassa      114 STVGVLVRGVPWDSVDNYTTAEAISN-VAKERNPEASIIRVRPWKRRDGESRG--------------------LLLVEVATASAACFLQDNLFLWDGGAYPCEPF-QASSNVQQCFRCWGIGHT-ARFCR
consensus        131 .  ....... .      . . .           ..    .. .           .                  . .  ..     ..  .  .   . . ..   ... ....*..*......  .*.

jockey_gag_Dmel  224 QDP-ICGKCSGP-HMTG-----FALCI---------------SDVCLCINCGG------DHVSTDK-SCPVRAEKAKKLKPRSRLPMTNNIATLKPPQRSSS-GYIPAEAL-------RTNISYADIARR
jockey_gag_Dfun  227 RDP-ICAKCAGS-HMTG-----SLLCT---------------SDICMCVNCGG------DHASTDK-DCPVRIEKLRKMKPSPRLPVPDNATNNKHNRASSARGFIPAEAV-------RGNMSYADIVRP
X-element_gag    215 KAH-ICVKCAGE-HP-------AKDCTRPR------------IELCTCYNCGGQ------HPANYK-GCSKLQAFLQRSRPRSGVAGRTEVSDRPTPRGLAGGKEIPSS---------RGGISYADVARG
L_dispar_gag     210 ARP-RCVKCLGD-HH-------TSQCERPKDISLC-------KEPPACVLCGEY-----GHPANYR-GCPRAPRRLVRQPNTNGKALYYNKTFVPAPLPTHNAWARP----------------LLNSKEA
Tribolium_gag    201 AEH-RCVKCGEA-HD-------TKVCTKES------------KEPPKCANCNGP------HTANYR-GCPQFPKLQKTATPRTTAPAKAAPTKAAACQGRHPQGSSPQGSS-----PQKGRRSQANSRPQ
Culex1_gag       203 LAA-LCVKCGEK-HF-------SADCRLPNKAELARADKSATREAIKCANCSGQ------HTANYR-GCP-----TRKNYLAKLAEKKAELRNVQPPTLRPFRNTVPQNGN---------------TVPQ
Culex_Cm_gag     206 LRP-RCVKCGES-HL-------SEACALPRKADLG-DKAEQTKPHVKCANCDGN------HTGNYR-GCV-----ARKAYLEEQEKRKKKAS--HPPQRS-TSAAVPAAGQR--------------TVPA
Helena_Dsim      211 RHP-NCARCGEN-HL-------TKLCVLPN------------DQQPICIHCGGN------HTASYK-GCQFYQEYLRRSMGTVKKQQPKQARFDKSATNQQQPQQKQQHAIASTPKDHTGCLSYADIARN
Doc_Drome        216 LKS-VCVVCSEP-HT-------TANCPKNKDD----------KSVKKCSNCGEK------HTANYR-GCVVYKELKSRLNKRIATAHTY------NKVNFYSPQPIFQPP-LTVPSTTPT-ISFASALKS
N_vitripennis    208 ANW-VCAFCAKD-HA-------TPACQKKDN----------KEIPPVCANCNGQ------HRATYR-GCPKAPKSPKTQ-KEPPQSSKN----NNSHPR------------FNAIKNTNTPTSYANAVTG
TART_C_gag       209 NPF-KCMKCGQL-HA-------TVSCTKPK------------NLPATCANCNGS------HVSSYK-GCP-AFQEAKQRLSINKIQSLHSQPTHLQTPRNKHPYPKPT---HLQTPRNK--QPYTHPLPR
HeT-A_Dyak       211 KPP-RCMKCAGQ-HW-------SSECTKPR------------STPATCSNCQGN------HISAYK-GCP-AYKAEKQKLAVNRIDFHKIRTIMDAKSNNNERQPRPP---FNKTPR----LPYS-----
HeT-A_Dm         212 RPP-RCMKCAGE-HL-------SSCCTKPR------------TTPATCVNCSGQ------HISAYK-GCP-AYKAEKQKLAANNVDINKIRTIKDATNNFY-KRQGPPL--RNNTPR----LPHSSAILS
Hebe_ORF1        211 TKQQVCSRCGEN-HS-------NDKCTVTD-------------DAVKCYNCKGN------HIATSK-ECSHYREQEKKMQNMVNQYATTSKQVTQAPS-IYNTQDFPPLSQFNQTQKQL--LTDN-----
B_mori_gag       215 STKIFCPKCGKH-HP---------NCETN---------------SFKCINCKGN------HMALAK-TCPIYLKERRIREIMSEFNCTYRK----A-SLMYVPPFSPAV---HRETSEN--ITYTMQNTT
Ifac_Dmel_gag    203 SVE-TCINCSETKHTND-----GEKCTNEK----------------NCLNCRNNPELDHQHSPIDR-KCPTFIKNQEL----TAIKTTQKVDHKTAQH-IYFERHGFQT-----------KNTYAKTLTN
Ifac_Dtes_gag    193 SPK-ICTNCSEV-HPAD-----DELCSKPK----------------FCSNCKFNTDILNNHSPIDK-NCTEFLKQKEI----TIIKTIEKVDFTTAKK-IYHQRNPHQS------------TPYAEVARH
BgI_B_glabrata   201 RNT-VCARCAGEGHE-------DKGCTAQF----------------KCPNCQAG------HSAYSK-DCPVWKQEVAV----QEYKARNGCTFSQAKS-AVLALP-KGQ--------FGLTKTYAQAVAK
Chaetomium       213 KEA-LCGRCGTGAHGEGGR-AGEALCPTQQG-----------QVPCKCPCCGGK------HPGWAK-ECPGRARAKKEAR----EA-YQYRPR-VFEPAR---------------------TAAAEPITA
Talaromyces      237 GKPPKCAICAGD-HEAIEHSCTVVDCRVGPAKP-------CKHTVIRCANCKGA------HEATSP-KCPKAREARQRAIRRMREQSLQDLIP-LDETFAVVPP-------------------------K
Ajellomyces      215 VNE-TCGYCAEP-HN-------TRDCRKREEDP---------NSTPKCALCKGP------HAAWSN-NCLTRQAEIAKVEQ-ARRNRPSYYIR-PDATSKVAQPATRP---------TIPLFTGGTLRQT
Tad_Ncrassa      221 QDD-ICARCGEAKH-EGDR-FGEVNCPSNDD-----------KSLVYCKPCGKK-----GHCAYNRKECPILRKAIAKASV-AHAERPRAFAPARTQPERCWSRRQ------------RLWTRVSCQTPP
consensus        261     .*..*..  *        .  *....                .* .* ..      * . .. .*.   .  ..                 .          .              ....  .
```

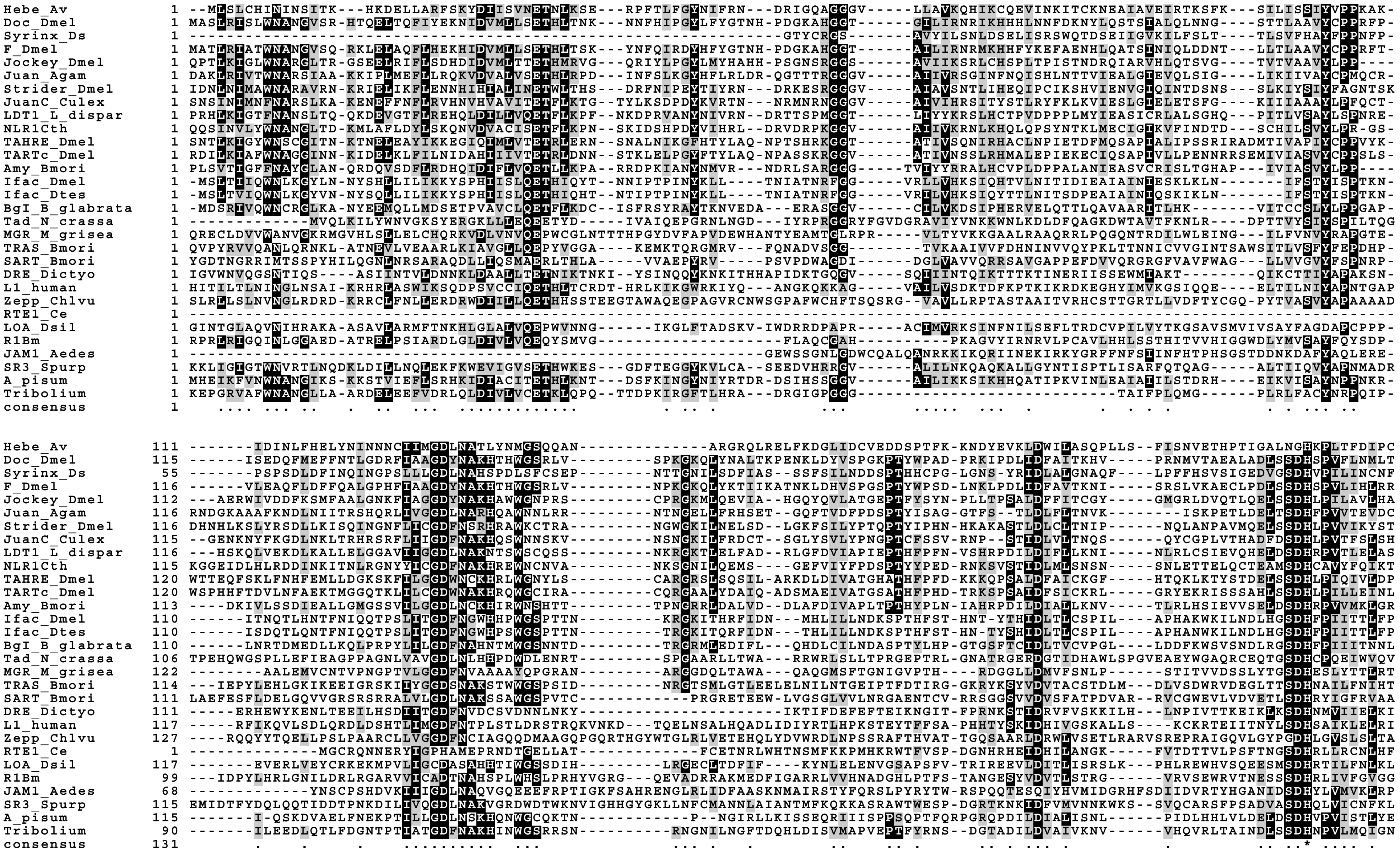

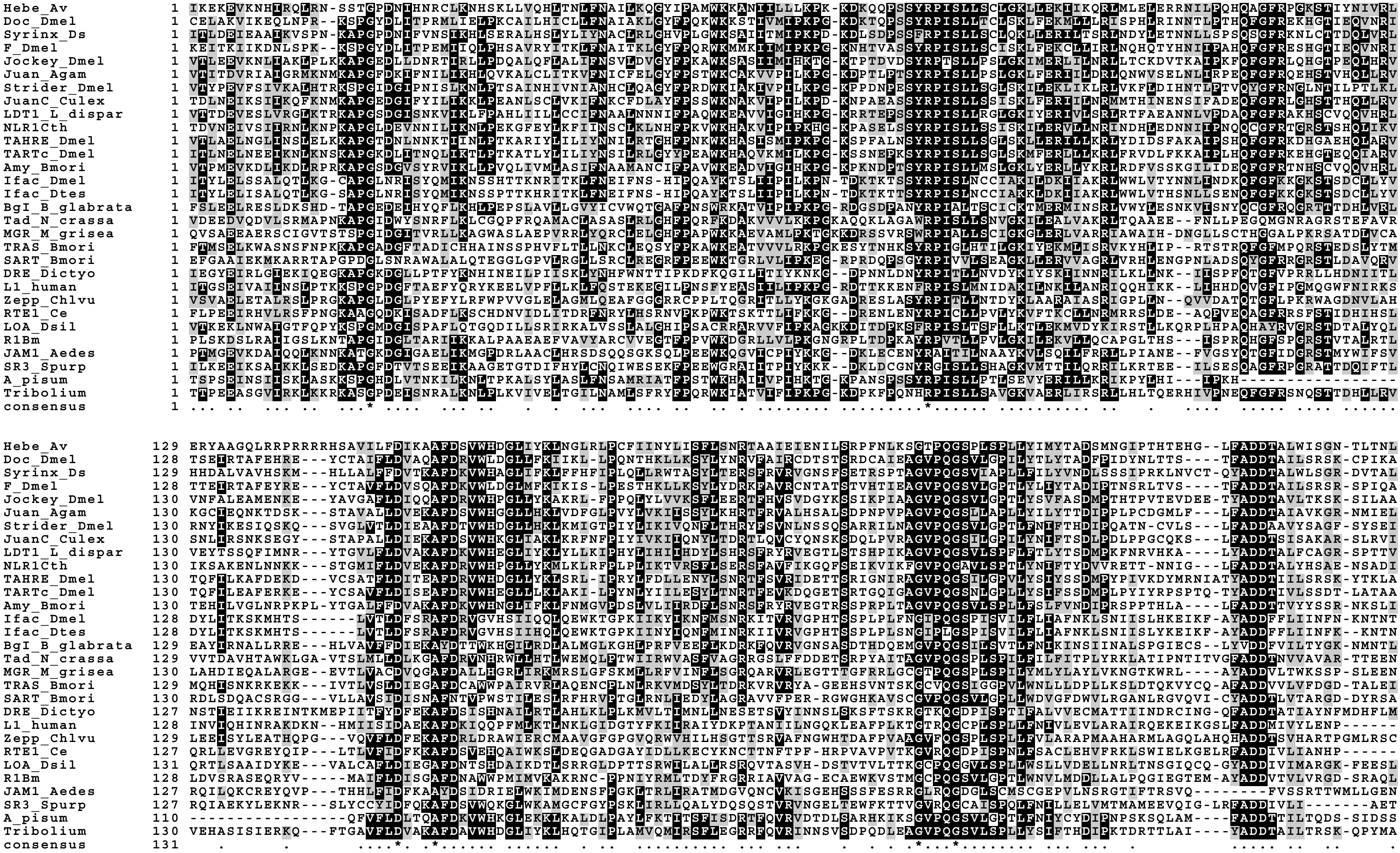
Hebe_Av 1
Doc_Dmel 1
Syrinx_Ds 1
F_Dmel 1
Jockey_Dmel 1
Juan_Agam 1
Strider_Dmel 1
JuanC_Culex 1
LDT1_L_dispar 1
NLR1Cth 1
TAHRE_Dmel 1
TARTc_Dmel 1
Amy_Bmori 1
Ifac_Dmel 1
Ifac_Dtes 1
BgI_B_glabrata 1
Tad_N_crassa 1
MGR_M_grisea 1
TRAS_Bmori 1
SART_Bmori 1
DRE_Dictyo 1
L1_human 1
Zepp_Chlvu 1
RTE1_Ce 1
LOA_Dsil 1
R1Bm 1
JAM1_Aedes 1
SR3_Spurp 1
A_pisum 1
Tribolium 1
consensus 1
Hebe_Av 129
Doc_Dmel 128
Syrinx_Ds 129
F_Dmel 128
Jockey_Dmel 130
Juan_Agam 130
Strider_Dmel 130
JuanC_Culex 130
LDT1_L_dispar 130
NLR1Cth 130
TAHRE_Dmel 130
TARTc_Dmel 130
Amy_Bmori 130
Ifac_Dmel 128
Ifac_Dtes 128
BgI_B_glabrata 129
Tad_N_crassa 129
MGR_M_grisea 130
TRAS_Bmori 129
SART_Bmori 130
DRE_Dictyo 127
L1_human 128
Zepp_Chlvu 129
RTE1_Ce 127
LOA_Dsil 131
R1Bm 128
JAM1_Aedes 127
SR3_Spurp 127
A_pisum 110
Tribolium 130
consensus 131
