# Supplementary material for: A subtelomeric non-LTR retrotransposon Hebe in the bdelloid rotifer Adineta vaga is subject to inactivation by deletions but not 5' truncations
Source: Mob DNA. 2010 Apr 1;1:12. doi: 10.1186/1759-8753-1-12 (PMC2861651; doi:10.1186/1759-8753-1-12)
Supplement: Additional file 2 — Nucleotide sequences of Hebe elements obtained in this study. [file 1759-8753-1-12-S2.PDF]

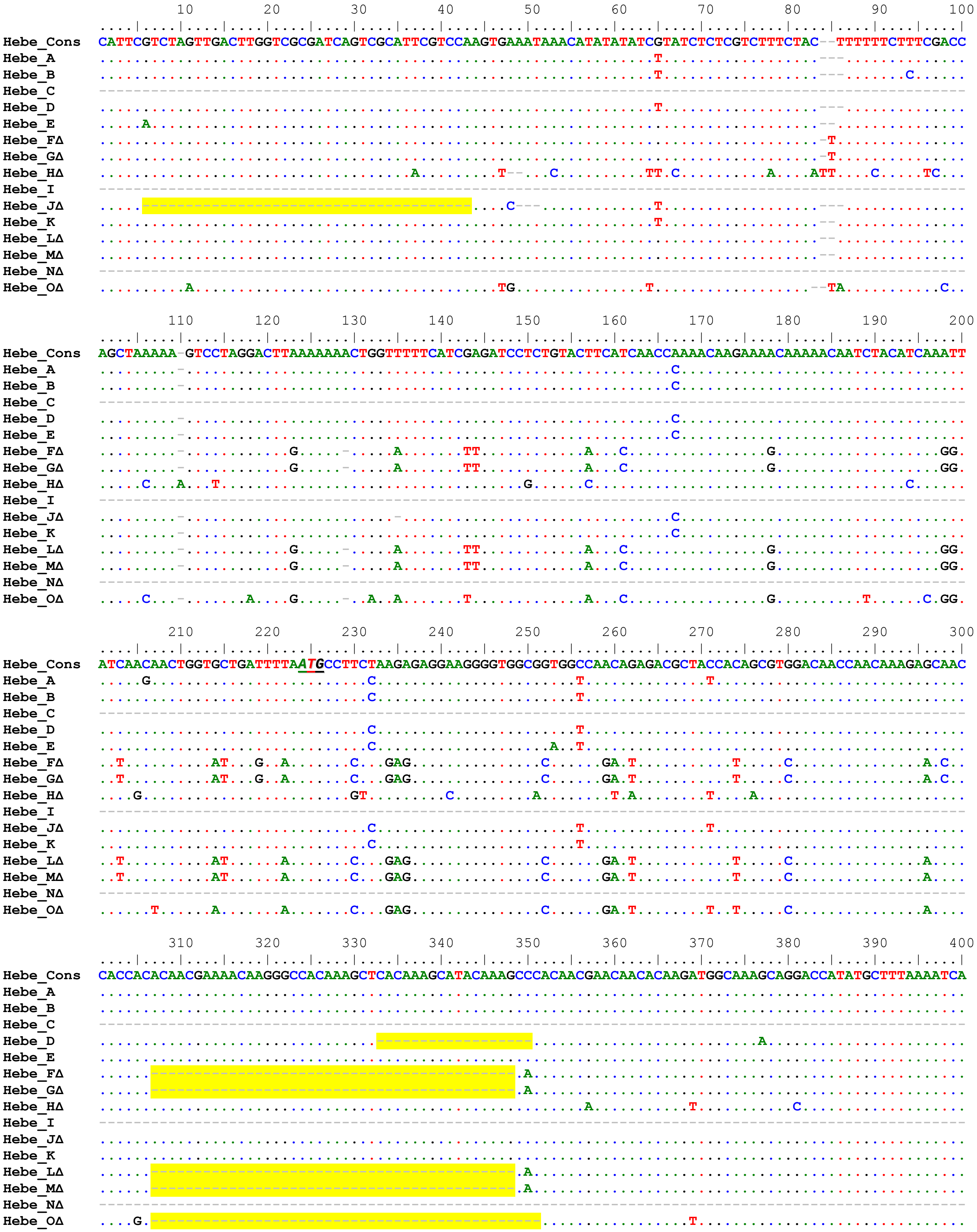
Hebe_Cons CATTCGTCTAGTTGACTTGGTCGCGATCAGTCGCATTCGTCCAAGTGAAATAAACATATATATCGTATCTCTCGTCTTTCTAC--TTTTTTCTTTCGACC
Hebe_Cons AGCTAAAAA-GTCCTAGGACTTAAAAAAACTGGTTTTTCATCGAGATCCTCTGTACTTCATCAACCAAAACAAGAAAACAAAAACAATCTACATCAAATT
Hebe_Cons ATCAACAACTGGTGCTGATTTTAATGCCTTCTAAGAGAGGAAGGGGTGGCGGTGGCCAACAGAGACGCTACCACAGCGTGGACAACCAACAAAGAGCAAC
Hebe_Cons CACCACACAACGAAAACAAGGGCCACAAAGCTCACAAAGCATACAAAGCCCACAACGAACAACACAAGATGGCAAAGCAGGACCATATGCTTTAAAATCA
Hebe_A
Hebe_B
Hebe_C
Hebe_D
Hebe_E
Hebe_FΔ
Hebe_GΔ
Hebe_HΔ
Hebe_I
Hebe_JΔ
Hebe_K
Hebe_LΔ
Hebe_MΔ
Hebe_NΔ
Hebe_OΔ

```
                    410       420       430       440       450       460       470       480       490       500
           ....|....|....|....|....|....|....|....|....|....|....|....|....|....|....|....|....|....|....|....|
Hebe_Cons  AGCCAACCAACATTTAGTATCGCCCCTCTTATTCTAGAGGGTGTTAACTTAAACAAGTTGCAGCTAAATGATATATTGAAGCAACATCTTGCTGAAGTCA
Hebe_A     ........................................................A...........................................
Hebe_B     ........................................................A...........................................
Hebe_C     ----------------------------------------------------------------------------------------------------
Hebe_D     ........................................................A...........................................
Hebe_E     .............................A..........................A...........................................
Hebe_FΔ    ..........A.........................................................................................
Hebe_GΔ    ..........A.........................................................................................
Hebe_HΔ    .................C........................................................T......A..................
Hebe_I     ----------------------------------------------------------------------------------------------------
Hebe_JΔ    ........................................................A...........................................
Hebe_K     ........................................................A...........................................
Hebe_LΔ    ..........A...............A.........................................................................
Hebe_MΔ    ..........A...............A.........................................................................
Hebe_NΔ    ----------------------------------------------------------------------------------------------------
Hebe_OΔ    ..........A................................................................G............A...........

                    510       520       530       540       550       560       570       580       590       600
           ....|....|....|....|....|....|....|....|....|....|....|....|....|....|....|....|....|....|....|....|
Hebe_Cons  ATATCCATGACATCCAACTTGGACGAAATGGAATATTCACATTATATGCAAGTGATGTTAAATCGTTCAACAACATATTAAACGACTTTTCATCAATATT
Hebe_A     ....................................................A...............................................
Hebe_B     ....................................................A...............................................
Hebe_C     ----------------------------------------------------------------------------------------------------
Hebe_D     ....................................................A...............................................
Hebe_E     ....................................................A.....C.........................................
Hebe_FΔ    ........................................................................................C...........
Hebe_GΔ    ........................................................................................C...........
Hebe_HΔ    .....................................T............................C.................................
Hebe_I     ----------------------------------------------------------------------------------------------------
Hebe_JΔ    ....................................................A...............................................
Hebe_K     ....................................................A...............................................
Hebe_LΔ    ..............G.........A...............................................................C...........
Hebe_MΔ    ..............G.........A...............................................................C...........
Hebe_NΔ    ----------------------------------------------------------------------------------------------------
Hebe_OΔ    .....T.......G...G...................T...C..............CCC........TT...TA...........T.C.A..GA....C.

                    610       620       630       640       650       660       670       680       690       700
           ....|....|....|....|....|....|....|....|....|....|....|....|....|....|....|....|....|....|....|....|
Hebe_Cons  ATCGTCAAACGGTCAACCATCAGCCACGGTATACGTTCCAAGATCTATACAAAGAATCAAGGACACAGAAAAGATCGCCTTCGTAAAAAGAGTCGATCTA
Hebe_A     ....................................................................................................
Hebe_B     ....................................................................................................
Hebe_C     ----------------------------------------------------------------------------------------------------
Hebe_D     ....................................................................................................
Hebe_E     ....................................................................................................
Hebe_FΔ    .............................................................................G......................
Hebe_GΔ    .............................................................................G......................
Hebe_HΔ    ..........A.............T.A......................................................................T.G
Hebe_I     ----------------------------------------------------------------------------------------------------
Hebe_JΔ    ....................................................................................................
Hebe_K     ............................................................A.......................................
Hebe_LΔ    .................T................C.................................................................
Hebe_MΔ    .................T................C.................................................................
Hebe_NΔ    ----------------------------------------------------------------------------------------------------
Hebe_OΔ    ....A...................G........T......C....A..T.............................G.....................

                    710       720       730       740       750       760       770       780       790       800
           ....|....|....|....|....|....|....|....|....|....|....|....|....|....|....|....|....|....|....|....|
Hebe_Cons  GAACTACCAAACGATCGAATAACTGAAGCACTAAAGAACGTTGGTCTTGAAGTAACAGAAGTTATTCGATTAACAAGCAAAGATGGTAAGACTCCAACAC
Hebe_A     ....................................................................................................
Hebe_B     ....................................................................................................
Hebe_C     ----------------------------------------------------------------------------------------------------
Hebe_D     ....................................................................................................
Hebe_E     ....................................................................................................
Hebe_FΔ    ...T................................................................................................
Hebe_GΔ    ...T................................................................................................
Hebe_HΔ    ...A....................A..............A.........................A...........................G......
Hebe_I     ----------------------------------------------------------------------------------------------------
Hebe_JΔ    ....................................................................................................
Hebe_K     ....................................................................................................
Hebe_LΔ    ...T..........................A.................C...................................................
Hebe_MΔ    ...T..........................A.................C...................................................
Hebe_NΔ    ----------------------------------------------------------------------------------------------------
Hebe_OΔ    ...T................................................................................................
```

```
                   810       820       830       840       850       860       870       880       890       900
           ....|....|....|....|....|....|....|....|....|....|....|....|....|....|....|....|....|....|....|....|
Hebe_Cons  GAACAGTCAAGATATCATTTAGCGATGCAACAAATCGAAATATCTTTGTGCAAACTGGTTTACAAGTGGATTGCATGCACTTCACCGCTGAACCAGCAAC
Hebe_A     .................................................................................................C..
Hebe_B     .................................................................................................G..
Hebe_C     ----------------------------------------------------------------------------------------------------
Hebe_D     .................................................................................................G..
Hebe_E     ........................................................................................C........G..
Hebe_FΔ    .....................................................................................A..A...........
Hebe_GΔ    .....................................................................................A..A...........
Hebe_HΔ    .T.....A..A...........T.................C.CG.........................................A..............
Hebe_I     ----------------------------------------------------------------------------------------------------
Hebe_JΔ    .................................................................................................C..
Hebe_K     .................................................................................................G..
Hebe_LΔ    .......................A.......C..C........G...............................C.........A..............
Hebe_MΔ    .......................A.......C..C........G...............................C.........A..............
Hebe_NΔ    ----------------------------------------------------------------------------------------------------
Hebe_OΔ    ................................T..........A.........................................A.T............

                   910       920       930       940       950       960       970       980       990      1000
           ....|....|....|....|....|....|....|....|....|....|....|....|....|....|....|....|....|....|....|....|
Hebe_Cons  ACAAAATTCCAAACCGGTGCAATGTTACATTTGCTTAAAATACAACCATGTAGCCAAATACTGCAAAACCAAGCAACAAGTATGTAGTCGATGTGGTGAA
Hebe_A     ....................................................................................................
Hebe_B     ..................T.................................................................................
Hebe_C     ----------------------------------------------------------------------------------------------------
Hebe_D     ....................................................................................................
Hebe_E     ....................................................................................................
Hebe_FΔ    ............................................................G.......................................
Hebe_GΔ    ............................................................G.......................................
Hebe_HΔ    .A.......A.................TG................TT..........G..............A.............T......C......
Hebe_I     ----------------------------------------------------------------------------------------------------
Hebe_JΔ    ....................................................................................................
Hebe_K     ....................................................................................................
Hebe_LΔ    .........................................G............G.............................................
Hebe_MΔ    .........................................G............G.............................................
Hebe_NΔ    ----------------------------------------------------------------------------------------------------
Hebe_OΔ    ....................................................................................................

                  1010      1020      1030      1040      1050      1060      1070      1080      1090      1100
           ....|....|....|....|....|....|....|....|....|....|....|....|....|....|....|....|....|....|....|....|
Hebe_Cons  AATCATAGCAACGACAAATGTACTGTTACAGATGATGCAGTCAAGTGCTACAACTGTAAAGGTAATCATATTGCTACTTCCAAAGAATGTTCACATTATA
Hebe_A     ...................................C................................................................
Hebe_B     ...................................C................................................................
Hebe_C     ----------------------------------------------------------------------------------------------------
Hebe_D     ...................................C................................................................
Hebe_E     ....................................................................................................
Hebe_FΔ    ....................................................................................................
Hebe_GΔ    ....................................................................................................
Hebe_HΔ    .....C....TG....T.....A.....A..................A...................................C..C.............
Hebe_I     ----------------------------------------------------------------------------------------------------
Hebe_JΔ    ...................................C................................................................
Hebe_K     ....................................................................................................
Hebe_LΔ    .........................................T..........................................................
Hebe_MΔ    .........................................T........................................G.................
Hebe_NΔ    ----------------------------------------------------------------------------------------------------
Hebe_OΔ    ....................................................................................................

                  1110      1120      1130      1140      1150      1160      1170      1180      1190      1200
           ....|....|....|....|....|....|....|....|....|....|....|....|....|....|....|....|....|....|....|....|
Hebe_Cons  GAGAACAAGAAAAGAAGATGCAAAACATGGTTAACCAATATGCAACAACAAGCAAACAAGTAACACAAGCACCATCAATCTACAACACACATGACTTCCC
Hebe_A     ....................................................................................G...............
Hebe_B     ...........................................................................................G........
Hebe_C     ----------------------------------------------------------------------------------------------------
Hebe_D     ....................................................................................G...............
Hebe_E     ....................................................................................................
Hebe_FΔ    ...........................................................................................G........
Hebe_GΔ    ...........................................................................................G........
Hebe_HΔ    ....G..T........A....TG..T...A...........T....................G..A.T........T..A....G....AG...T.AT..
Hebe_I     ----------------------------------------------------------------------------------------------------
Hebe_JΔ    ....................................................................................G...............
Hebe_K     ....................................................................................................
Hebe_LΔ    .............................A........A.........................................................C...
Hebe_MΔ    .............................A........A.........................................................C...
Hebe_NΔ    ----------------------------------------------------------------------------------------------------
Hebe_OΔ    ............................................................................................A.......
```

```
                1210      1220      1230      1240      1250      1260      1270      1280      1290      1300
           ....|....|....|....|....|....|....|....|....|....|....|....|....|....|....|....|....|....|....|....|
Hebe_Cons  ACCTCTATCACAATTCAACCAAACACAAAAACAACTATTAACGGACAATTTATTCGACGATATCATTAATGCTCTTACCAGCAAAATGGAAACCATCATC
Hebe_A     ....................................................................................................
Hebe_B     ....................................................................................................
Hebe_C     ----------------------------------------------------------------------------------------------------
Hebe_D     ....................................................................................................
Hebe_E     ....................................................................................................
Hebe_FΔ    ....................................................................................................
Hebe_GΔ    ....................................................................................................
Hebe_HΔ    ..A.T..........TG.....C.......G....A......A....G.C.......T.....TC....C.....C..T.....G.......AA......
Hebe_I     ----------------------------------------------------------------------------------------------------
Hebe_JΔ    ....................................................................................................
Hebe_K     .........................................................T..........................................
Hebe_LΔ    ....................................................................................................
Hebe_MΔ    ....................................................................................................
Hebe_NΔ    ----------------------------------------------------------------------------------------------------
Hebe_OΔ    ....................................................................................................

                1310      1320      1330      1340      1350      1360      1370      1380      1390      1400
           ....|....|....|....|....|....|....|....|....|....|....|....|....|....|....|....|....|....|....|....|
Hebe_Cons  GAAAAAACTACCAATCGACTATTCAAAGCACTACATCAGAAGATCAAGAAGATCGAAAAATCATTCGGACTCCTCGATCGTAATAATGAAGAAAAATATA
Hebe_A     ....................................................................................................
Hebe_B     ........................................................................A...........................
Hebe_C     ----------------------..............................................................................
Hebe_D     ....................................................................................................
Hebe_E     ....................................................................................................
Hebe_FΔ    ........................................................................A...........................
Hebe_GΔ    ........................................................................A...........................
Hebe_HΔ    A...C....G........T............................................A.T...............T..G...........A...
Hebe_I     ----------------------------------------------------------------------------------------------------
Hebe_JΔ    ....................................................................................................
Hebe_K     ....................................................................................................
Hebe_LΔ    ....................................................................................................
Hebe_MΔ    ....................................................................................................
Hebe_NΔ    ----------------------------------------------------------------------------------------------------
Hebe_OΔ    ....................................................................................................

                1410      1420      1430      1440      1450      1460      1470      1480      1490      1500
           ....|....|....|....|....|....|....|....|....|....|....|....|....|....|....|....|....|....|....|....|
Hebe_Cons  TATTAACTGATTCAGACTCTGATTCCAACGAACAAAGTCAAGCAGTTAAGATCATCAAACCAAAACCAAAAAAACAACAGAGTGAAGCTGACAAATCGTC
Hebe_A     ....................................................................................................
Hebe_B     .................................................................................A..................
Hebe_C     ....................................................................................................
Hebe_D     ....................................................................................................
Hebe_E     ....................................................................................................
Hebe_FΔ    .................................................................................A..................
Hebe_GΔ    .................................................................................A..................
Hebe_HΔ    .C..............................G....C...........A..T......T.T.....T...---.......A..................
Hebe_I     ----------------------------------------------------------------------------------------------------
Hebe_JΔ    ....................................................................................................
Hebe_K     ....................................................................................................
Hebe_LΔ    ....................................................................................................
Hebe_MΔ    ....................................................................................................
Hebe_NΔ    ----------------------------------------------------------------------------------------------------
Hebe_OΔ    .................................................................................A.......A..........

                1510      1520      1530      1540      1550      1560      1570      1580      1590      1600
           ....|....|....|....|....|....|....|....|....|....|....|....|....|....|....|....|....|....|....|....|
Hebe_Cons  TACAACAGAGAATGTCACTTCAAATAAACCTACTATGCCATCAACTCCTACTACATCAAAAGACGCACAAAATAAAAAAGAAGGATCCAATACAAACAAG
Hebe_A     ......................................................G...................C.........................
Hebe_B     .........................G..........................................................................
Hebe_C     ......................................................G.............................................
Hebe_D     ......................................................G...................C.........................
Hebe_E     ......................................................G.............................................
Hebe_FΔ    .........................G..........................................................................
Hebe_GΔ    .........................G..........................................................................
Hebe_HΔ    C......TC..C.......................CAATT......T..................A.G....AC................AG...C....
Hebe_I     ----------------------------------------------------------------------------------------------------
Hebe_JΔ    ......................................................G...................C.........................
Hebe_K     ...................................................C................................................
Hebe_LΔ    .....................................T........T..........................................C..........
Hebe_MΔ    .....................................T........T..........................................C..........
Hebe_NΔ    ----------------------------------------------------------------------------------------------------
Hebe_OΔ    C..C..........................................T.................A.........................CG....T...
```

```
                 1610      1620      1630      1640      1650      1660      1670      1680      1690      1700
             ....|....|....|....|....|....|....|....|....|....|....|....|....|....|....|....|....|....|....|....|
Hebe_Cons    AGAGCCCTTTCTTCCAACAACTCTCCTGATGCATCTATAAACGACAGCAAAACACTTAAAACAAGCAGCAACGATGCTTAGTTTATGCCATATCAACATT
Hebe_A       ..............................................AG....................................................
Hebe_B       ..............................................A.....................................................
Hebe_C       ..............................................AG..............T.....................................
Hebe_D       ..............................................AG....................................................
Hebe_E       ..............................................AG..............T.....................................
Hebe_FΔ      ..............................................A.....................................................
Hebe_GΔ      ..............................................A.....................................................
Hebe_HΔ      ...AT.T.C...C......GT....T...CA........C.T.CA......GAT.............A........A...A............T......
Hebe_I       ----------------------------------------------------------------------------------------------------
Hebe_JΔ      ..............................................AG....................................................
Hebe_K       ..............................................A.....................................................
Hebe_LΔ      ..G................................C.............................T.A........T.......................
Hebe_MΔ      ..G................................C.............................T.A........T.......................
Hebe_NΔ      ----------------------------------------------------------------------------------------------------
Hebe_OΔ      ...AAT...C..C....................................................T.A................................

                 1710      1720      1730      1740      1750      1760      1770      1780      1790      1800
             ....|....|....|....|....|....|....|....|....|....|....|....|....|....|....|....|....|....|....|....|
Hebe_Cons    AACTCAATTACCAAACACAAAGATGAACTCCTAGCCAGATTCTCCAAATACGATATTATCTCTGTTAATGAAACTAATCTAAAGAGCGAAAGACCATTCA
Hebe_A       ....................................................................................................
Hebe_B       ....................................................................................................
Hebe_C       ....................................................................................................
Hebe_D       ....................................................................................................
Hebe_E       ....................................................................................................
Hebe_FΔ      ....................................................................................................
Hebe_GΔ      ....................................................................................................
Hebe_HΔ      ........C..G.................T.............................T....................G.........C.........
Hebe_I       ----------------------------------------------------------------------------------------------------
Hebe_JΔ      ....................................................................................................
Hebe_K       ....................................................................................................
Hebe_LΔ      ....................................................................................................
Hebe_MΔ      ....................................................................................................
Hebe_NΔ      ----------------------------------------------------------------------------------------------------
Hebe_OΔ      ..................................A..........................T......................................

                 1810      1820      1830      1840      1850      1860      1870      1880      1890      1900
             ....|....|....|....|....|....|....|....|....|....|....|....|....|....|....|....|....|....|....|....|
Hebe_Cons    CTCTTTTTGGTTATAACATCTTCAGAAATGATCGAATAGGACAAGCTGGGGGTGGAGTATTACTAGCAGTGAAACAACATATCAAGTGTCAAGAAGTACT
Hebe_A       .G..................................................................................................
Hebe_B       ....................................................................................................
Hebe_C       ....................................................................................................
Hebe_D       .................................................T............T.....................................
Hebe_E       ....................................................................................................
Hebe_FΔ      ....................................................................................................
Hebe_GΔ      ....................................................................................................
Hebe_HΔ      .....C.............T.............................T............T.....................................
Hebe_I       ----------------------------------------------------------------------------------------------------
Hebe_JΔ      .G..................................................................................................
Hebe_K       .................................................T............T...................................A.
Hebe_LΔ      .......G......................................................A.....................................
Hebe_MΔ      .......G......................................................A.....................................
Hebe_NΔ      ----------------------------------------------------------------------------------------------------
Hebe_OΔ      A...................................................................................................

                 1910      1920      1930      1940      1950      1960      1970      1980      1990      2000
             ....|....|....|....|....|....|....|....|....|....|....|....|....|....|....|....|....|....|....|....|
Hebe_Cons    AAACAAAATAACCTGCAAGAATGAAGCGATAGCAGTAGAGATTCGAACTAAATCATTCAAATCAATACTAATATCCTCCATTTACGTACCACCAAAAGCA
Hebe_A       ........C...........................................................................................
Hebe_B       G...................................................................................................
Hebe_C       ........C...........................................................................................
Hebe_D       ....................................................................................................
Hebe_E       ........C...........................................................................................
Hebe_FΔ      G...................................................................................................
Hebe_GΔ      G...................................................................................................
Hebe_HΔ      ....G...C.......................................A.........................................G.........
Hebe_I       ----------------------------------------------------------------------------------------------------
Hebe_JΔ      ........C...........................................................................................
Hebe_K       ....................................................................................................
Hebe_LΔ      G...................................................................................................
Hebe_MΔ      G...................................................................................................
Hebe_NΔ      ----------------------------------------------------------------------------------------------------
Hebe_OΔ      ....G...C.........................................................................C.................
```

```
                 2010      2020      2030      2040      2050      2060      2070      2080      2090      2100
           ....|....|....|....|....|....|....|....|....|....|....|....|....|....|....|....|....|....|....|....|
Hebe_Cons  AAGATCGATATCAACTTATTCCACGAACTTTATAACATTAACAACAACTGCATTATCATGGGTGATCTTAATGCAACATTATATAATATGGGATCACAAC
Hebe_A     ..............................................G.C..........................................................
Hebe_B     ....................................................................................................
Hebe_C     ....................................G.C..................................A..........................
Hebe_D     .....................T.................................................A...........C................
Hebe_E     ....................................G.C.............................................................
Hebe_FΔ    ....................................................................................................
Hebe_GΔ    ....................................................................................................
Hebe_HΔ    ....................................................................................................
Hebe_I     ----------------------------------------------------------------------------------------------------
Hebe_JΔ    ....................................G.C.............................................................
Hebe_K     ....................................................................................................
Hebe_LΔ    ......................G....................G........................................................
Hebe_MΔ    ......................G....................G........................................................
Hebe_NΔ    ----------------------------------------------------------------------------------------------------
Hebe_OΔ    ........C...........................................................................................

                 2110      2120      2130      2140      2150      2160      2170      2180      2190      2200
           ....|....|....|....|....|....|....|....|....|....|....|....|....|....|....|....|....|....|....|....|
Hebe_Cons  AAGCTAATGCTAGAGGAAGACAGCTGCAAGAATTATTTAAAGATGGTCTTATTGATTGTGTCGACGATGATAGTCCAACTTTCGAAAAAAATGATTATGA
Hebe_A     ....................................................................................................
Hebe_B     ....................................................................................................
Hebe_C     ....................................................................................................
Hebe_D     ....................T...............................................................................
Hebe_E     ....................................................................................................
Hebe_FΔ    ....................................................................................................
Hebe_GΔ    ....................................................................................................
Hebe_HΔ    ....................................................................................................
Hebe_I     ----------------------------------------------------------------------------------------------------
Hebe_JΔ    ....................................................................................................
Hebe_K     ...........................G....................................A..................A................
Hebe_LΔ    ....................................................................................................
Hebe_MΔ    ....................................................................................................
Hebe_NΔ    ----------------------------------------------------------------------------------------------------
Hebe_OΔ    ...................G.........................C.........A......A...................T.....G.........A.

                 2210      2220      2230      2240      2250      2260      2270      2280      2290      2300
           ....|....|....|....|....|....|....|....|....|....|....|....|....|....|....|....|....|....|....|....|
Hebe_Cons  AGTTAAACTAGATTGGATTCTAGCAAGTCAACCACTTCTTTCATTCATATCAAACGTTGAGACTCATCCAACAATCGGTGCATTAAATGGCCATAAACCA
Hebe_A     ....................................................................................................
Hebe_B     ..............................................G.....................................................
Hebe_C     ....................................................................................................
Hebe_D     ....................................................................................................
Hebe_E     ....................................................................................................
Hebe_FΔ    ...........................................C........................................................
Hebe_GΔ    ...........................................C........................................................
Hebe_HΔ    ....................................................................................................
Hebe_I     ----------------------------------------------------------------------------------------------------
Hebe_JΔ    ....................................................................................................
Hebe_K     ....................................................................................................
Hebe_LΔ    ....................................................................................................
Hebe_MΔ    ....................................................................................................
Hebe_NΔ    ----------------------------------------------------------------------------------------------------
Hebe_OΔ    ......................A..................................C..A.......................G..C..T.....G...

                 2310      2320      2330      2340      2350      2360      2370      2380      2390      2400
           ....|....|....|....|....|....|....|....|....|....|....|....|....|....|....|....|....|....|....|....|
Hebe_Cons  TTAACATTTGATATTCCTTGCGGAGCTGAACCCAAACCGGCTTCGTCAAGAATTTCATTTAATTTCAAAGCAGCAAAATGGTCAAAATTTAGGTGCAAGT
Hebe_A     .......................................................T............................................
Hebe_B     .......................................................T............................................
Hebe_C     ....................................................................................................
Hebe_D     ............................................................................................A.......
Hebe_E     ....................................................................................................
Hebe_FΔ    ............................................................................................A.......
Hebe_GΔ    ............................................................................................A.......
Hebe_HΔ    ............................................................................................A.......
Hebe_I     ----------------------------------------------------------------------------------------------------
Hebe_JΔ    .......................................................T............................................
Hebe_K     ....................................................................................................
Hebe_LΔ    .................................................A..................................................
Hebe_MΔ    .................................................A..................................................
Hebe_NΔ    ----------------------------------------------------------------------------------------------------
Hebe_OΔ    C.................G.T............G......C.A..C..............C............-------..------------------
```

```
                  2410      2420      2430      2440      2450      2460      2470      2480      2490      2500
            ....|....|....|....|....|....|....|....|....|....|....|....|....|....|....|....|....|....|....|....|
Hebe_Cons   TAGATCAACAACTGATGCTGTGGAAAAATGATCATCATTTAGATTCAACAGCAGACATAGAAGAATATACATCATTCATTACCACTAGTATACTAGAAGC
Hebe_A      ....................................................................................................
Hebe_B      .............................A......................................................................
Hebe_C      ....................................................................................................
Hebe_D      ...............................................G......................T.............................
Hebe_E      ....................................................................................................
Hebe_FΔ     ...............................................G......................T.............................
Hebe_GΔ     ...............................................G......................T.............................
Hebe_HΔ     ....................................................................................................
Hebe_I      ----------------------------------------------------------------------------------------------------
Hebe_JΔ     ....................................................................................................
Hebe_K      ................................................T................C..................................
Hebe_LΔ     ...............................................G......................T.............................
Hebe_MΔ     ...............................................G......................T.............................
Hebe_NΔ     ----------------------------------------------------------------------------------------------------
Hebe_OΔ     -----------------------------------------------------.................G.....T.......A.......T.GA....

                  2510      2520      2530      2540      2550      2560      2570      2580      2590      2600
            ....|....|....|....|....|....|....|....|....|....|....|....|....|....|....|....|....|....|....|....|
Hebe_Cons   AACAAAAGAAGCCGTTCCACAAACAAAGCAGATGATCCGAACGTATACACCAAGTGAAGTATCGATAAGCCTGATAAAACAAAAACATCAAGCATATCGA
Hebe_A      ....................................................................................................
Hebe_B      ....................................................................................................
Hebe_C      ....................................................................................................
Hebe_D      ..................G....A............................................................................
Hebe_E      ....................................................................................................
Hebe_FΔ     ....................................................................................................
Hebe_GΔ     ....................................................................................................
Hebe_HΔ     ....................................................................................................
Hebe_I      ----------------------------------------------------------------------------------------------------
Hebe_JΔ     ....................................................................................................
Hebe_K      ....................................................................................................
Hebe_LΔ     .................................................................A..................................
Hebe_MΔ     ..T..............................................................A..................................
Hebe_NΔ     ----------------------------------------------------------------------------------------------------
Hebe_OΔ     ............T.............................................TC........................................

                  2610      2620      2630      2640      2650      2660      2670      2680      2690      2700
            ....|....|....|....|....|....|....|....|....|....|....|....|....|....|....|....|....|....|....|....|
Hebe_Cons   AAATGGAAGAAGACTGGAAACAACTTAGATAAACATCTATATTACAATTCCAAAGTCTTGCTTACAAATTCACTTAGAAACGACAGAAGAAATAACTTCA
Hebe_A      ....................................................................................................
Hebe_B      ..................................................T.................................................
Hebe_C      .............................................T......................................................
Hebe_D      .......G............................................................................................
Hebe_E      ....................................................................................................
Hebe_FΔ     .................................................T............C.....................................
Hebe_GΔ     .................................................T............C.....................................
Hebe_HΔ     ................A.....................T....................A.....C.........C....T..........GC.....TC
Hebe_I      ----------------------------------------------------------------------------------------------------
Hebe_JΔ     ....................................................................................................
Hebe_K      ....................................................................................................
Hebe_LΔ     ....................................................................................................
Hebe_MΔ     ....................................................................................................
Hebe_NΔ     ----------------------------------------------------------------------------------------------------
Hebe_OΔ     ..............A........GAC...........A........T...........AA....................T...........C..T..T.

                  2710      2720      2730      2740      2750      2760      2770      2780      2790      2800
            ....|....|....|....|....|....|....|....|....|....|....|....|....|....|....|....|....|....|....|....|
Hebe_Cons   ACAAGTTAATGTCATCTTTATGCCATAAGAAAATGTATTCGGACAAAGTTTGGCTGACGGTGCGCAAGTTCCACAACAAAAGGATCAAGCAAACCTACGC
Hebe_A      ....................................................................................................
Hebe_B      ............................................................G.......................................
Hebe_C      .........................C..........................................................................
Hebe_D      .....................................................T..............................................
Hebe_E      .....................................................T..............................................
Hebe_FΔ     ......................T.............................................................................
Hebe_GΔ     ......................T.............................................................................
Hebe_HΔ     ................C.....T..C...........C.......................A..............T.....................T.
Hebe_I      ----------------------------------------------------------------------------------------------------
Hebe_JΔ     ....................................................................................................
Hebe_K      ..................................................................G.................................
Hebe_LΔ     ......................T.............................................................................
Hebe_MΔ     ......................T.............................................................................
Hebe_NΔ     ----------------------------------------------------------------------------------------------------
Hebe_OΔ     ......................T.G.........A.....A....................A..T...........T....................T..
```

```
                 2810      2820      2830      2840      2850      2860      2870      2880      2890      2900
           ....|....|....|....|....|....|....|....|....|....|....|....|....|....|....|....|....|....|....|....|
Hebe_Cons  CAACATCATGAAATACAACAATACAACTGCAACATCAGACAAGGAGAAAGCAGACTTATTTGCAGATTACTTCGAAAATGAAGTCTACTCTCACACTGCT
Hebe_A     ....................................................................................................
Hebe_B     ....................................................................................................
Hebe_C     ....................................................................................................
Hebe_D     ..........................................................................G.........................
Hebe_E     ....................................................................................................
Hebe_FΔ    ....................................................................................................
Hebe_GΔ    ....................................................................................................
Hebe_HΔ    T...G..................T...A.................................T......................AC.......A......
Hebe_I     ----------------------------------------------------------------------------------------------------
Hebe_JΔ    ....................................................................................................
Hebe_K     .C..................................................................................................
Hebe_LΔ    ....................................................................................................
Hebe_MΔ    ....................................................................................................
Hebe_NΔ    ------------------------------------------------------------------------------------.......GTT......
Hebe_OΔ    .......................T.....................................C..........T..........................G

                 2910      2920      2930      2940      2950      2960      2970      2980      2990      3000
           ....|....|....|....|....|....|....|....|....|....|....|....|....|....|....|....|....|....|....|....|
Hebe_Cons  GATACGTTGCCACTTCATGATCAAGTAACACGCCAAGCAAACAAAGTCAAAAGTAAAACTATAACCTCTTCAAACACACCAAAGTGGAAGCAAATCAAAG
Hebe_A     ....................................................................................................
Hebe_B     ....................................................................................................
Hebe_C     ....................................................................................................
Hebe_D     ..............................................................................T.....................
Hebe_E     ..............................................................................T.....................
Hebe_FΔ    ....................................................................................................
Hebe_GΔ    ....................................................................................................
Hebe_HΔ    ...G.AC...G.T.................T.............CA.......C...CA....G....A....................A.T...T....
Hebe_I     --------------------------------....................................................................
Hebe_JΔ    ....................................................................................................
Hebe_K     ........................................................................G...........................
Hebe_LΔ    ....................................................................................................
Hebe_MΔ    ....................................................................................................
Hebe_NΔ    .....A......T.................A.T.................G.A.GGT.A.........AA........T.T.G.......TT..CT....
Hebe_OΔ    ................................................................T..A...G............................

                 3010      3020      3030      3040      3050      3060      3070      3080      3090      3100
           ....|....|....|....|....|....|....|....|....|....|....|....|....|....|....|....|....|....|....|....|
Hebe_Cons  AAAAAGAAGTCAAAAATCATATAAGACAACTTAGAAATAGCTCCACTGGTCCAGACAACATTCATAACCGATGTTTGAAGAATCATTCAAAATTACTGGT
Hebe_A     ....................................................................................................
Hebe_B     ....................................................................................................
Hebe_C     ....................................................................................................
Hebe_D     ....................................................................................................
Hebe_E     ....................................................................................................
Hebe_FΔ    .................................................................................................T..
Hebe_GΔ    .................................................................................................T..
Hebe_HΔ    T.G.............C.......................G........................................................T..
Hebe_I     ....................................................................................................
Hebe_JΔ    ....................................................................................................
Hebe_K     ....................................................................................................
Hebe_LΔ    .................................................................................................TA.
Hebe_MΔ    .................................................................................................TA.
Hebe_NΔ    T.G..........CT.................C.............A.....G..A.....C......A.G.....A..A...A.......G.....T..
Hebe_OΔ    ............T.....................................T.............C...............................GT..

                 3110      3120      3130      3140      3150      3160      3170      3180      3190      3200
           ....|....|....|....|....|....|....|....|....|....|....|....|....|....|....|....|....|....|....|....|
Hebe_Cons  ACAGCATCTAACAAATCTGTTTAATGCAATTTTGAAACAAGGTTATATTCCAGCAATGTGGAAAAAGGCTAATATTATTCTTCTATTAAAGCCAAAGAAA
Hebe_A     ....................................................................................................
Hebe_B     ....................................................................................................
Hebe_C     ....................................................................................................
Hebe_D     ....................................................................................................
Hebe_E     ....................................................................................................
Hebe_FΔ    ...................................................................................................G
Hebe_GΔ    ...................................................................................................G
Hebe_HΔ    ..............................---------------------------..........................................G
Hebe_I     ....................................................................................................
Hebe_JΔ    ....................................................................................................
Hebe_K     ....................................................................................................
Hebe_LΔ    ................................................................................................A..G
Hebe_MΔ    ................................................................................................A..G
Hebe_NΔ    .....................C.................................................................G...A.......G
Hebe_OΔ    ............................................................................................TC.....G
```

```
                3210      3220      3230      3240      3250      3260      3270      3280      3290      3300
           ....|....|....|....|....|....|....|....|....|....|....|....|....|....|....|....|....|....|....|....|
Hebe_Cons  GACAAACAACAACCGTCTAGCTATCGACCGATTAGTCTCCTTAGTTGCTTAGGCAAACTATTGGAGAAAATAATCAAACAACGTTTAATGCTCGAACTTG
Hebe_A     ....................................................................................................
Hebe_B     ....................................................................................................
Hebe_C     ....................................................................................................
Hebe_D     ....................................................................................................
Hebe_E     ....................................................................................................
Hebe_FΔ    ....................T...............................................................................
Hebe_GΔ    ....................T...............................................................................
Hebe_HΔ    ....................T...............................................................................
Hebe_I     ....................................................................................................
Hebe_JΔ    ................................................C...................................................
Hebe_K     ....................................................................................................
Hebe_LΔ    A...................T...............................................................................
Hebe_MΔ    A...................T...............................................................................
Hebe_NΔ    ....................T...............................................................................
Hebe_OΔ    ..........------------------------------------------------------------------------....G........G....

                3310      3320      3330      3340      3350      3360      3370      3380      3390      3400
           ....|....|....|....|....|....|....|....|....|....|....|....|....|....|....|....|....|....|....|....|
Hebe_Cons  AACGACGAAACATCTTACCACAACATCAAGCCGGATTTAGACCAGGTAAAAGCACTATATACAACATCGTGCGATTAGAAAGATACGCTGCAGGACAACT
Hebe_A     ....................................................................................................
Hebe_B     ....................................................................................................
Hebe_C     ....................................................................................................
Hebe_D     ....................................................................................................
Hebe_E     ....................................................................................................
Hebe_FΔ    .......T..................................................................................A.........
Hebe_GΔ    .......T..................................................................................A.........
Hebe_HΔ    .......T............A...............................................................................
Hebe_I     ....................................................................................................
Hebe_JΔ    ....................................................................................................
Hebe_K     ....................................................................................................
Hebe_LΔ    .......T............................................................................................
Hebe_MΔ    .......T............................................................................................
Hebe_NΔ    .......T............................................................................................
Hebe_OΔ    ....................................................................................................

                3410      3420      3430      3440      3450      3460      3470      3480      3490      3500
           ....|....|....|....|....|....|....|....|....|....|....|....|....|....|....|....|....|....|....|....|
Hebe_Cons  TAGACGACCACGTCGACGACGTCACTCAGCTGTCATTCTTTTCGACATCAAAGCCGCATTTGATTCGGTATGGCACGATGGTTTGATATACAAGCTTAAT
Hebe_A     ....................................................................................................
Hebe_B     ....................................................................................................
Hebe_C     ....................................................................................................
Hebe_D     ....................................................................................................
Hebe_E     ....................................................................................................
Hebe_FΔ    ......................................................A.............................................
Hebe_GΔ    ......................................................A.............................................
Hebe_HΔ    C...............................C............T........A.....C.....T.................A...............
Hebe_I     ....................................................................................................
Hebe_JΔ    ....................................................................................................
Hebe_K     ....................................................................................................
Hebe_LΔ    ......................................................A.................................A...........
Hebe_MΔ    ......................................................A.................................A...........
Hebe_NΔ    ..------------........................................A.............................................
Hebe_OΔ    .............................................TG.......A...........T.................................

                3510      3520      3530      3540      3550      3560      3570      3580      3590      3600
           ....|....|....|....|....|....|....|....|....|....|....|....|....|....|....|....|....|....|....|....|
Hebe_Cons  GATCTACGTCTTCCCTGTTTCATCATCAACTATCTGATCTCATTCTTAAGCAATAGAACGGCTGCAATTGAAATCGAAAATATATTATCACGTCCGTTTA
Hebe_A     ....................................................................................................
Hebe_B     ....................................................................................................
Hebe_C     ....................................................................................................
Hebe_D     ....................................................................................................
Hebe_E     ....................................................................................................
Hebe_FΔ    ....................................................................................................
Hebe_GΔ    ....................................................................................................
Hebe_HΔ    ...................................................................................................T
Hebe_I     ...............................................................A....................................
Hebe_JΔ    ....................................................................................................
Hebe_K     .G..................................................................................................
Hebe_LΔ    .............................................................T.....................................T
Hebe_MΔ    .............................................................T.....................................T
Hebe_NΔ    ....................................................................................................
Hebe_OΔ    .................................TC.........T..G..T......................G..G..................A....
```

```
                3610      3620      3630      3640      3650      3660      3670      3680      3690      3700
          ....|....|....|....|....|....|....|....|....|....|....|....|....|....|....|....|....|....|....|....|
Hebe_Cons ATCTGAAGAGCGGTACACCACAAGGATCTCCGTTATCACCATTACTTTATATTATGTATACGGCAGATTCGATGAATGGAATACCAACTCATACAGAACA
Hebe_A    ....................................................................................................
Hebe_B    ....................................................................................................
Hebe_C    ....................................................................................................
Hebe_D    ....................................................................................................
Hebe_E    ....................................................................................................
Hebe_FΔ   ....................................................................................................
Hebe_GΔ   ....................................................................................................
Hebe_HΔ   ..............................................................C.....................................
Hebe_I    ....................................................................................................
Hebe_JΔ   ....................................................................................................
Hebe_K    .....................................T..............................................................
Hebe_LΔ   .........................................................................C..........................
Hebe_MΔ   .........................................................................C..........................
Hebe_NΔ   ....................................................................................................
Hebe_OΔ   ...........A........T...............................C...............................................

                3710      3720      3730      3740      3750      3760      3770      3780      3790      3800
          ....|....|....|....|....|....|....|....|....|....|....|....|....|....|....|....|....|....|....|....|
Hebe_Cons TGGGTTGTTCGCCGACGACACTGCATTATGGACATCTGGCAACACCTTAACAAATCTTAACACCAGATTACAACAATCCATAGATGCATTCGAAAGTTGG
Hebe_A    ....................................................................................................
Hebe_B    ....................................................................................................
Hebe_C    ....................................................................................................
Hebe_D    ....................................................................................................
Hebe_E    ....................................................................................................
Hebe_FΔ   ..........................................T.........................................................
Hebe_GΔ   ..........................................T.........................................................
Hebe_HΔ   ..............................................................................T.....................
Hebe_I    ....................................................................................................
Hebe_JΔ   ....................................................................................................
Hebe_K    ................................T...................................................................
Hebe_LΔ   ....................................................................................................
Hebe_MΔ   ....................................................................................................
Hebe_NΔ   ....................................................................................................
Hebe_OΔ   ............T............C.............T............................................................

                3810      3820      3830      3840      3850      3860      3870      3880      3890      3900
          ....|....|....|....|....|....|....|....|....|....|....|....|....|....|....|....|....|....|....|....|
Hebe_Cons TGCAAGTCATGGAAATTAAAACTTCAACCAACTAAGACAGAGCTGATACACTTCAGCATACATCCAAGAAAACACTACAAGAATCCAGTGAAAGTTAAAG
Hebe_A    ....................................................................................................
Hebe_B    ....................................................................................................
Hebe_C    ....................................................................................................
Hebe_D    ....................................................................................................
Hebe_E    ....................................................................................................
Hebe_FΔ   ....................................................................................................
Hebe_GΔ   ....................................................................................................
Hebe_HΔ   ....................................................................................................
Hebe_I    ....................................................................................................
Hebe_JΔ   ....................................................................................................
Hebe_K    ....................................................................................................
Hebe_LΔ   ....................C.....................................................A....................G....
Hebe_MΔ   ....................C.....................................................A....................G....
Hebe_NΔ   ....................................................................................................
Hebe_OΔ   .......................................................A.......A.....G....G.........................

                3910      3920      3930      3940      3950      3960      3970      3980      3990      4000
          ....|....|....|....|....|....|....|....|....|....|....|....|....|....|....|....|....|....|....|....|
Hebe_Cons TTGAAGACACCATCATCAAACCACTGGACTCCACACGTTACTTGGGTGTAATTATAGATAAAAGATTAAAATGGCGAGCACATCTCGAACATATTGAAAG
Hebe_A    ....................................................................................................
Hebe_B    ....................................................................................................
Hebe_C    ....................................................................................................
Hebe_D    ....................................................................................................
Hebe_E    ......................T.............................................................................
Hebe_FΔ   ............................T.......................................................................
Hebe_GΔ   ............................T.......................................................................
Hebe_HΔ   .............................................A...............................A................C.....
Hebe_I    ....................................................................................................
Hebe_JΔ   ....................................................................................................
Hebe_K    ....................................................................................................
Hebe_LΔ   ............................T..T....................................................................
Hebe_MΔ   ....................................................................................................
Hebe_NΔ   ............................T.............................C.........................................
Hebe_OΔ   ............................T...............................C.................................C.....
```

```
                     4010      4020      4030      4040      4050      4060      4070      4080      4090      4100
           ....|....|....|....|....|....|....|....|....|....|....|....|....|....|....|....|....|....|....|....|
Hebe_Cons  CAAGATTGCTCCTCGGATCGGTCTACTTCGATATCTTTCGAGAACAGCATATGAACCCAACAACAAGACGATGATAAATATTTTCAAATCAATTGTACGC
Hebe_A     ....................................................................................................
Hebe_B     ....................................................................................................
Hebe_C     ....................................................................................................
Hebe_D     ....................................................................................................
Hebe_E     ...............................................................T....................................
Hebe_FΔ    ....................................................................................................
Hebe_GΔ    ....................................................................................................
Hebe_HΔ    .............A..........T..........G..............TC............................................G...
Hebe_I     ....................................................................................................
Hebe_JΔ    ....................................................................................................
Hebe_K     ..............................................A.....................................................
Hebe_LΔ    ..................T.................................................................................
Hebe_MΔ    ..................T.................................................................................
Hebe_NΔ    ....................T.............A.................................................................
Hebe_OΔ    ....................................................................................................

                     4110      4120      4130      4140      4150      4160      4170      4180      4190      4200
           ....|....|....|....|....|....|....|....|....|....|....|....|....|....|....|....|....|....|....|....|
Hebe_Cons  ACAGTTATCATTTATGGACATCCTATTCTCCTTACTGCTGATCAAAAAGTATGGGACCGTTTACAAATCATGCAGAATAAAGCAATTAGAGCTGCACTCG
Hebe_A     ....................................................................................................
Hebe_B     ....................................................................................................
Hebe_C     ....................................................................................................
Hebe_D     ....................................................................................................
Hebe_E     ....................................................................................................
Hebe_FΔ    .............................T...................C.......................C..........G...............
Hebe_GΔ    .............................T...................C.......................C..........G...............
Hebe_HΔ    ..................................A.................................................................
Hebe_I     ....................................................................................................
Hebe_JΔ    ....................................................................................................
Hebe_K     .......................................................................A............................
Hebe_LΔ    ..................................T.................................................................
Hebe_MΔ    ..................................T.................................................................
Hebe_NΔ    ...........C........................................................................................
Hebe_OΔ    ..............................................................................................G.....

                     4210      4220      4230      4240      4250      4260      4270      4280      4290      4300
           ....|....|....|....|....|....|....|....|....|....|....|....|....|....|....|....|....|....|....|....|
Hebe_Cons  GACTACCCATATATACATCTGTCAAATATATCCACAAGATCAGCAACATTCCAAAAATCAAAGATTATGCAATAGGACTACTTAAACATACACTTCAAAA
Hebe_A     ....................................................................................................
Hebe_B     ..................................................................................C.................
Hebe_C     ....................................................................................................
Hebe_D     ....................................................................................................
Hebe_E     ....................................................................................................
Hebe_FΔ    ...................................................................................................C
Hebe_GΔ    ...................................................................................................C
Hebe_HΔ    ....G.............................................T.....................A.....................A....C
Hebe_I     ..................................................................................C.................
Hebe_JΔ    ....................................................................................................
Hebe_K     ....................................................................................................
Hebe_LΔ    ..........G.................................................................G......................C
Hebe_MΔ    ..........G.................................................................G......................C
Hebe_NΔ    ....................................................................................................
Hebe_OΔ    ..A......................G................A.........................................................

                     4310      4320      4330      4340      4350      4360      4370      4380      4390      4400
           ....|....|....|....|....|....|....|....|....|....|....|....|....|....|....|....|....|....|....|....|
Hebe_Cons  GGCAACAACAAACAACGACATTACACTGAAGAATCATCTACAATGCATCTTAGACAAAATTTAAAAAA-GAAATCACCAACCATTACATATAATAGTAAC
Hebe_A     ....................................................................-.............................G.
Hebe_B     ....................................................................-...............................
Hebe_C     ....................................................................A...............................
Hebe_D     ....................................................................-...............................
Hebe_E     ....................................................................A...............................
Hebe_FΔ    .............................................T.....G..............---..........-.GT.................
Hebe_GΔ    .............................................T.....G..............---..........-.GT.................
Hebe_HΔ    C.................................................................---..........-.GCC..T...G....T....
Hebe_I     ....................................................................A...............................
Hebe_JΔ    ....................................................................-...............................
Hebe_K     ....................................................................A...............................
Hebe_LΔ    ....................................................C.A.
Hebe_MΔ    ....................................................C.A.
Hebe_NΔ    ....................................................................A...............................
Hebe_OΔ    ....................................................................-...............................
```

```
                4410      4420      4430      4440      4450      4460      4470      4480      4490      4500
            ....|....|....|....|....|....|....|....|....|....|....|....|....|....|....|....|....|....|....|....|
Hebe_Cons   ATCGAGAGAATAGACTATTCTTGTGTCAGTCGAATCGATGTAAAAAACGATATATCGCAAT-CAAATATACTATTCAGCTACTTCGTTTAACTCGTTCTT
Hebe_A      .............................................................-......................T...............
Hebe_B      .............................................................-......................................
Hebe_C      .............................................................-......................................
Hebe_D      .............................................................-......................................
Hebe_E      .............................................................-......................................
Hebe_FΔ     .............................................................-........T.............................
Hebe_GΔ     .............................................................-........T.............................
Hebe_HΔ     .......A.CA.T..C.............C......C.
Hebe_I      .............................................................-....................................G.
Hebe_JΔ     .............................................................-......................................
Hebe_K      ..........................T..................................-......................................
Hebe_LΔ
Hebe_MΔ
Hebe_NΔ     ....................................TG.AC.....T..........A.GCA..T...AG........T.............C.......
Hebe_OΔ     .............................................................-......................................

                4510      4520      4530      4540      4550      4560      4570      4580      4590      4600
            ....|....|....|....|....|....|....|....|....|....|....|....|....|....|....|....|....|....|....|....|
Hebe_Cons   ATACAAAATAAGAAGTCGATTCAATCTATATGCTGTATCGTTCTTTCGTTCTATTCAGTTATTTATTGATCGCATTCTTATATATAATATAATATCGAGC
Hebe_A      ....................................................................................................
Hebe_B      ....................................................................................................
Hebe_C      ....................................................................................................
Hebe_D      ....................................................................................................
Hebe_E      ....................................................................................................
Hebe_FΔ     .......................................
Hebe_GΔ     ....................................................................................................
Hebe_HΔ
Hebe_I      ............................................................................................C.......
Hebe_JΔ     ....................................................................................................
Hebe_K      ....................................................................................................
Hebe_LΔ
Hebe_MΔ
Hebe_NΔ     ..............A..........T...C........................C.......A................G....C....CG........T
Hebe_OΔ     ......................................................C.......A.................................T..T

                4610      4620      4630      4640      4650      4660      4670      4680      4690      4700
            ....|....|....|....|....|....|....|....|....|....|....|....|....|....|....|....|....|....|....|....|
Hebe_Cons   CAATCTTTAAACCGATTCATGTATACAGCAAATTCTTTTACTCGTTCTTATATACAATATGATATCGAGTCAACGTCTTTGCTGATCCTTACAATATCCA
Hebe_A      ....................................................................................................
Hebe_B      ....................................................................................................
Hebe_C      ................C..............................................................G....................
Hebe_D      ....................................................................................................
Hebe_E      ................C..............................................................G....................
Hebe_FΔ
Hebe_GΔ     ....................................................................................................
Hebe_HΔ
Hebe_I      ................C..............................................................G....................
Hebe_JΔ     ....................................................................................................
Hebe_K      ....................................................................................................
Hebe_LΔ
Hebe_MΔ
Hebe_NΔ     ...CG.C.-----------------------------..G..............T...............G.....A...AA....T.--.AGTC.....
Hebe_OΔ     ............T...........C.......................G...........T.A........G.T.AA.A.A.C...T.--.AGTC.A...

                4710      4720      4730      4740      4750      4760      4770      4780      4790      4800
            ....|....|....|....|....|....|....|....|....|....|....|....|....|....|....|....|....|....|....|....|
Hebe_Cons   GTAAATTCCTTGCTCGTTCTTATATACAATATGATATCGAGTCAACGTCTTTGCTGATCCTTACAATATCCAGTAAATTCCTTGCTCGTTCTTATATACA
Hebe_A      ....................................................................................................
Hebe_B      ....................................................................................................
Hebe_C      ........T..........................................G............................--------------------
Hebe_D      ........--------------------------------------------------------------------------------------------
Hebe_E      ........T..........................................G............................--------------------
Hebe_FΔ
Hebe_GΔ     ........T..........................G............................................T...................
Hebe_HΔ
Hebe_I      ......................C............G........................C...................T...................
Hebe_JΔ     ....................................................................................................
Hebe_K      ........--------------------------------------------------------------------------------------------
Hebe_LΔ
Hebe_MΔ
Hebe_NΔ     .CC..A..T-------------------------------------------------------------------------------------------
Hebe_OΔ     ........--------------------------------------------------------------------------------------------
```

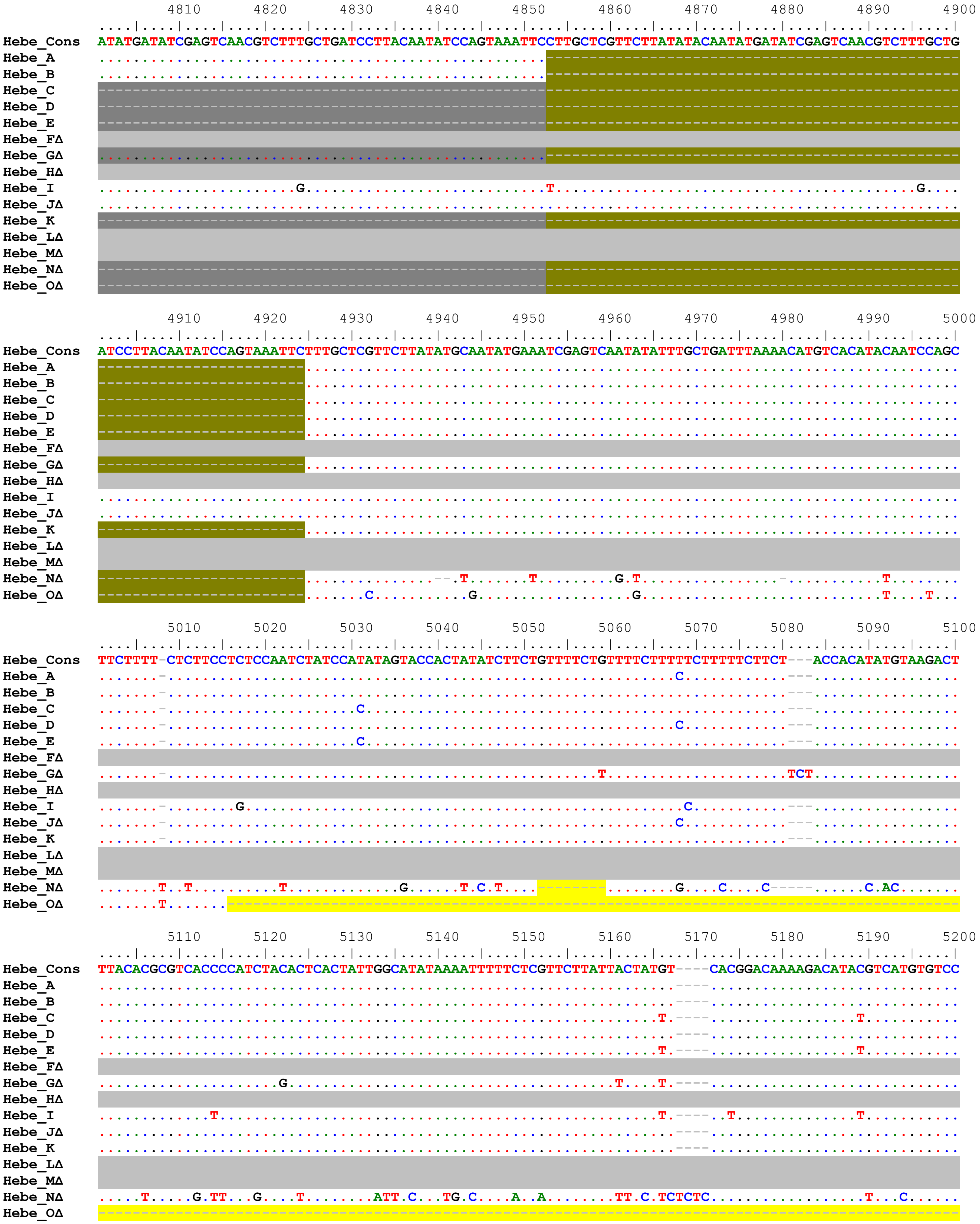
4810 4820 4830 4840 4850 4860 4870 4880 4890 4900
Hebe_Cons ATATGATATCGAGTCAACGTCTTTGCTGATCCTTACAATATCCAGTAAATTCCTTGCTCGTTCTTATATACAATATGATATCGAGTCAACGTCTTTGCTG
4910 4920 4930 4940 4950 4960 4970 4980 4990 5000
Hebe_Cons ATCCTTACAATATCCAGTAAATTCTTTGCTCGTTCTTATATGCAATATGAAATCGAGTCAATATATTTGCTGATTTAAAACATGTCACATACAATCCAGC
5010 5020 5030 5040 5050 5060 5070 5080 5090 5100
Hebe_Cons TTCTTTT-CTCTTCCTCTCCAATCTATCCATATAGTACCACTATATCTTCTGTTTTCTGTTTTCTTTTTCTTTTTCTTCT---ACCACATATGTAAGACT
5110 5120 5130 5140 5150 5160 5170 5180 5190 5200
Hebe_Cons TTACACGCGTCACCCCATCTACACTCACTATTGGCATATAAAATTTTTCTCGTTCTTATTACTATGT----CACGGACAAAAGACATACGTCATGTGTCC
Hebe_A
Hebe_B
Hebe_C
Hebe_D
Hebe_E
Hebe_FΔ
Hebe_GΔ
Hebe_HΔ
Hebe_I
Hebe_JΔ
Hebe_K
Hebe_LΔ
Hebe_MΔ
Hebe_NΔ
Hebe_OΔ

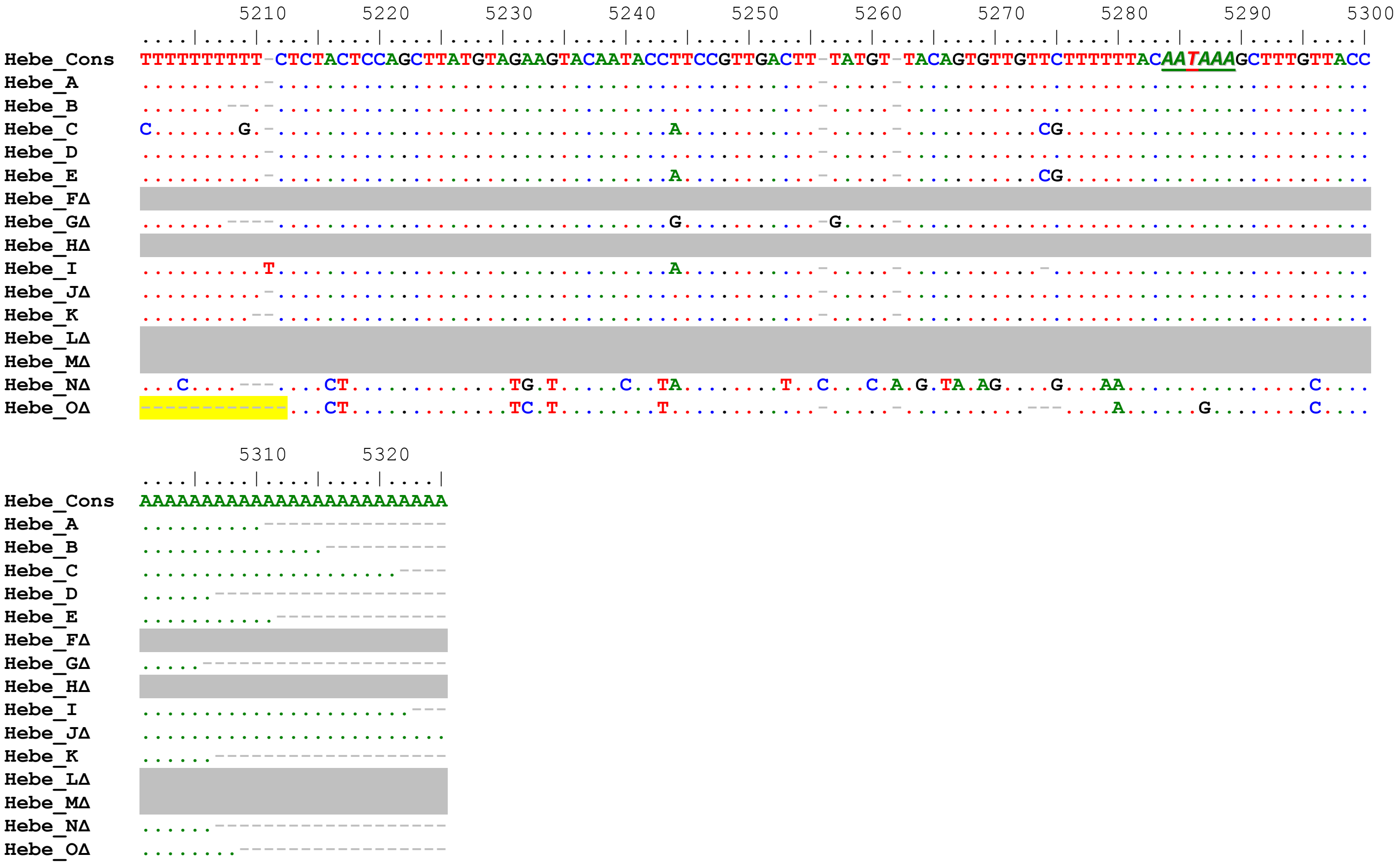

Supplementary Fig. 1. Nucleotide sequence alignment of *Hebe* copies depicted in Fig. 1. Dots indicate identity to the consensus sequence. Internal deletions are highlighted in yellow, and 3' terminal deletions in light gray. Additional copies of the 72-bp tandem repeat in the 3' UTR are highlighted in green and dark gray. The ATG codon and polyadenylation signal are underlined. Copies with deletions are marked by Δ.
