# Supplementary material for: A subtelomeric non-LTR retrotransposon Hebe in the bdelloid rotifer Adineta vaga is subject to inactivation by deletions but not 5' truncations
Source: Mob DNA. 2010 Apr 1;1:12. doi: 10.1186/1759-8753-1-12 (PMC2861651; doi:10.1186/1759-8753-1-12)
Supplement: Additional file 3 — Analysis of non-synonymous to synonymous substitution ratios in open reading frame (ORF) 1 and ORF2 of Hebe. [file 1759-8753-1-12-S3.PDF]

<u>Table S1</u>. Pairwise Ka/Ks values for ORF1 from different *Hebe* copies. Intact copies (A, B, E) are underlined. Incomplete (C, I, N) or nearly-identical (J) copies were not included in the analysis.

	**<u>A</u>**	**<u>B</u>**	D	**<u>E</u>**	F,G	H	K	L	M	O
**<u>A</u>**		0.44	0.15	0.06	0.30	0.46	0.16	0.43	0.45	0.46
**<u>B</u>**			1.05	0.15	0.18	0.45	0.29	0.46	0.48	0.43
D				0.14	0.34	0.47	0.38	0.48	0.49	0.48
**<u>E</u>**					0.22	0.42	0.03	0.34	0.35	0.38
F,G						0.46	0.26	0.64	0.67	0.43
H							0.47	0.44	0.45	0.46
K								0.41	0.43	0.42
L									N.A.	0.50
M										0.52
O										

<u>Table S2</u>. Pairwise Ka/Ks values for ORF2 from different *Hebe* copies. Intact copies (A, B, E) are underlined. Copy J, which is nearly-identical to copy A, was not included in the analysis.

	**<u>A</u>**	**<u>B</u>**	C	D	**<u>E</u>**	F,G	H	I	K	L	M	N	O
**<u>A</u>**		0.39	0.36	0.36	0.14	0.22	0.22	0.57	0.78	0.39	0.45	0.27	0.29
**<u>B</u>**			0.81	0.42	0.43	0.26	0.24	N.A.	0.97	0.46	0.52	0.26	0.30
C				0.44	0.24	0.24	0.24	0.57	0.98	0.43	0.49	0.27	0.29
D					0.28	0.25	0.26	1.15	1.06	0.42	0.48	0.25	0.29
**<u>E</u>**						0.20	0.22	0.24	0.66	0.37	0.41	0.23	0.27
F,G							0.27	0.18	0.38	0.87	0.92	0.41	0.33
H								0.27	0.31	0.28	0.30	0.36	0.31
I									0.86	0.30	0.33	0.25	0.34
K										0.55	0.61	0.31	0.34
L											0.28	0.45	0.37
M												0.45	0.37
N													0.35
O													
